# Influence of Maternal Lifestyle and Diet on Perinatal DNA Methylation Signatures Associated With Childhood Arterial Stiffness at 8 to 9 Years

**DOI:** 10.1161/HYPERTENSIONAHA.121.17396

**Published:** 2021-07-19

**Authors:** Robert Murray, Negusse Kitaba, Elie Antoun, Philip Titcombe, Sheila Barton, Cyrus Cooper, Hazel M. Inskip, Graham C. Burdge, Pamela A. Mahon, John Deanfield, Julian P. Halcox, Elizabeth A. Ellins, Jennifer Bryant, Charles Peebles, Karen Lillycrop, Keith M. Godfrey, Mark A. Hanson

**Affiliations:** 1 From the School of Human Development and Health, Institute of Developmental Sciences Building, Faculty of Medicine (R.M., N.K., E.A., G.C.B., K.M.G., M.A.H.), University of Southampton, United Kingdom; 2 MRC Lifecourse Epidemiology Unit (P.T., S.B., C.C., H.M.I., P.A.M., K.M.G.), University of Southampton, United Kingdom; 3 Centre for Biological Sciences, Faculty of Natural and Environmental Sciences (E.A., K.L.), University of Southampton, United Kingdom; 4 NIHR Southampton Biomedical Research Centre, University of Southampton and University Hospital Southampton NHS Foundation Trust, United Kingdom (H.M.I., K.L., K.M.G., M.A.H.); 5 Swansea University Medical School, Swansea University, United Kingdom (J.P.H., E.A.E.); 6 Department of Cardiac Magnetic Resonance Imaging, National Heart Centre Singapore (J.B.); 7 Wessex Cardiothoracic Centre, Southampton University Hospitals NHS Trust, United Kingdom (C.P.); 8 Institute of Cardiovascular Sciences, University College London, United Kingdom (J.D.).

**Keywords:** aorta, blood pressure, DNA methylation, epigenome, genetic variation

## Abstract

Increases in aortic pulse wave velocity, a measure of arterial stiffness, can lead to elevated systolic blood pressure and increased cardiac afterload in adulthood. These changes are detectable in childhood and potentially originate in utero, where an adverse early life environment can alter DNA methylation patterns detectable at birth. Here, analysis of epigenome-wide methylation patterns using umbilical cord blood DNA from 470 participants in the Southampton’s Women’s Survey identified differential methylation patterns associated with systolic blood pressure, pulse pressure, arterial distensibility, and descending aorta pulse wave velocity measured by magnetic resonance imaging at 8 to 9 years. Perinatal methylation levels at 16 CpG loci were associated with descending aorta pulse wave velocity, with identified CpG sites enriched in pathways involved in DNA repair (*P*=9.03×10^−11^). The most significant association was with cg20793626 methylation (within protein phosphatase, Mg2+/Mn2+ dependent 1D; β=−0.05 m/s/1% methylation change, [95% CI, −0.09 to −0.02]). Genetic variation was also examined but had a minor influence on these observations. Eight pulse wave velocity-linked dmCpGs were associated with prenatal modifiable risk factors, with cg08509237 methylation (within palmitoyl-protein thioesterase-2) associated with maternal oily fish consumption in early and late pregnancy. Lower oily fish consumption in early pregnancy modified the relationship between methylation and pulse wave velocity, with lower consumption strengthening the association between cg08509237 methylation and increased pulse wave velocity. In conclusion, measurement of perinatal DNA methylation signatures has utility in identifying infants who might benefit from preventive interventions to reduce risk of later cardiovascular disease, and modifiable maternal factors can reduce this risk in the child.

## Abstract 2

Supplemental Digital Content is available in the text.

**See Editorial, pp 801–803**

Cardiovascular disease (CVD) is a leading cause of death worldwide and the single largest cause of noncommunicable disease deaths.^1^ Early markers of CVD risk are important to identify individuals at particular risk who may benefit from preventive interventions. Increased arterial stiffness, which augments systolic blood pressure, increases cardiac afterload and leads to hemodynamic dysfunction, is a strong predictor of cardiovascular events and all-cause mortality^2,3^ and contributes significantly to the global chronic CVD burden.^4^ Raised aortic pulse wave velocity (aPWV), the most widely used measure of increased arterial stiffness,^3,5,6^ is positively associated with carotid and aortic plaque formation, calcification, and coronary atherosclerosis.^7,8^ Longitudinal studies have shown that increased aPWV is one of the earliest detectable measures of vascular pathology^9^ and is a significant independent risk factor for future clinical CVD.^5,10,11^

While clinical presentation of CVD occurs primarily in later life, this is preceded by subclinical changes in both cardiovascular structure and function. Many of these changes can be detected in childhood,^12,13^ consistent with the thesis that CVD risk may partly originate in utero.^14^ Modifiable prenatal risk factors such as maternal body mass index (BMI) and gestational weight gain, smoking, as well as dietary components such as vitamin D, folic acid, and consumption of oily fish, the main dietary source of the polyunsaturated fatty acids, eicosapentaenoic acid (EPA), and docosahexaenoic acid (DHA),^15–17^ have been shown to influence later CVD risk markers such as carotid intima-media thickness,^18^ aPWV,^19^ and elevated blood pressure (BP)^20^ in children.

One mechanism by which the early life environment has been suggested to induce persistent changes in offspring phenotype is through altered epigenetic regulation of gene expression.^21,22^ Studies in both humans and animals have reported that aspects of the prenatal environment are associated with altered DNA methylation and subsequent disease risk.^23–25^ For example, we previously showed that DNA methylation at birth at CpG loci within the promoter of ANRIL, a long noncoding RNA with strong genetic links to CVD,^26^ was associated with arterial stiffness in children aged 8 to 9 years^27^; furthermore, maternal cholesterol levels have been correlated with early fetal atherogenesis and methylation of key genes in fetal cholesterol metabolism.^28^ Taken together, these studies examining candidate genes suggest that it is possible to identify epigenetic markers in early life that are predictive of later CVD risk. A systematic approach to identify alterations throughout the methylome could allow earlier risk stratification and provide insights into modifiable prenatal factors that may attenuate such risk, providing a basis for developing future interventions. To determine whether differential methylation of CpG loci at birth is associated with CVD risk factors in childhood, we performed the first comprehensive analysis of the relationship between epigenome-wide DNA methylation, measured at birth in umbilical cord blood DNA, and detailed assessment of cardiovascular structure and function in the children at age 8 to 9 years, using data from the UK Southampton Women’s Survey (SWS). The relationship between differentially methylated CpG loci associated with CVD risk and fixed genetic differences as well as modifiable factors, specifically maternal adiposity and diet during pregnancy, was then examined to explore whether modifiable lifestyle factors during pregnancy could influence the child’s methylation of these marks at birth and thus potentially their CVD risk in later childhood.

## Methods

Anonymized data and materials have been made publicly available at the Gene Expression Omnibus under accession number GSE154915 and can be accessed at https://www.ncbi.nlm.nih.gov/geo/.

### Study Cohort

The SWS is an ongoing prospective cohort study of 12 583, initially nonpregnant, women aged 20 to 34 years living in the city of Southampton, United Kingdom.^2^ Assessments of lifestyle, diet,^30,31^ and anthropometry were performed at study entry (April 1998–December 2002). Women who subsequently became pregnant were followed through pregnancy and their offspring were followed up during infancy and childhood. Here, we focus on data from the cardiovascular follow-up of a sub-sample of the children aged 8 to 9 years.

### Maternal Characteristics, Diet, Micronutrient Status, and Plasma Fatty Acid Composition

Full details of the ascertainment of maternal BMI, weight gain in pregnancy, smoking, dietary intake assessment, serum vitamin D, and vitamin B12 status are available in the Data Supplement. Measurements of maternal plasma EPA and DHA during pregnancy were available in a subset of individuals in whom DNA methylation was measured (early pregnancy, n=316; late pregnancy, n=377). As described previously,^32^ the concentrations of DHA and EPA were calculated from the ratio of their peak areas to the peak area of the internal standard, multiplied by the amount of standard and corrected for the volume of plasma extracted.

### Cardiovascular Measures in the SWS Cohort

Magnetic resonance imaging pulse wave velocity (aPWV), arterial distensibility, BP, carotid intima-media thickness, flow-mediated dilatation (FMD), and reactive hyperemia were measured in the children at 8 to 9 years of age. Full details are available in the Data Supplement.

### DNA Extraction

Genomic DNA was extracted from the buffy coat of umbilical cord blood samples using the QIAamp Blood DNA mini kit (Qiagen). Quality of the genomic DNA was assessed by agarose gel electrophoresis, and quantity of genomic DNA was checked on the NanoDrop ND-1000 (NanoDrop Technologies).

### Measurement of DNA Methylation

DNA from cord blood was bisulfite converted using the Zymo EZ DNA Methylation-Gold kit. The Infinium Human MethylationEPIC BeadChip array (Illumina Inc, San Diego, CA) was used to analyze DNA methylation levels. Quality control, filtering, and normalization of the methylation data are described in the Data Supplement.^33–42^ Probes located on SNPs, on the X or Y chromosome and cross-reactive probes were discarded.

### Genotyping

SNP genotyping was performed using Human OminiExpress-24v1.2 at Edinburgh Clinical Research Facility. Genotyping call was analyzed in Illumina Genome Studio 2.0.2 using genotyping Module 2.0.2 following the manufacturer technical note.^43^ PLINK version 1.9 beta^44^ was used for SNP data management and quality control.

### Statistical Analysis

Regression models using limma^41^ were run with methylation as the outcome variable. All models included the following as covariates: maternal smoking, child’s sex, age at measurements, batch effect, position on chip and the predicted values for B cell, CD4 T cell, CD8 T cell, monocyte, natural killer cell, and nucleated red blood cell counts. The analysis was controlled for multiple testing with the Benjamini-Hochberg adjustment for false discovery rate (FDR). Linear regression was used to obtain an estimate of phenotypic variance explained by multiple dmCpGs, alongside other model components, associated with aPWV. The 16 dmCpG sites associated with aPWV (FDR adjusted *P* value ≤0.05) were included in a linear regression model alongside covariates listed above. The aim of the model was to optimize the variance explained in the outcome; to do so, the least significant dmCpG was removed from the model and the estimate for phenotypic variance explained by the model was examined. This process was repeated until the removal of a dmCpG would cause a reduction in the total variance explained by the model.

To investigate the influence of SNPs on methylation, GEM^45^ Bioconductor/R package was applied with a filter for ≥0.05 minor allele frequency and Bonferroni correction for multiple testing. To determine the relationship between genetic variation and methylation levels identified CpG sites, multivariate regression was performed, controlling for array batch, position, blood cell components, sex of child, and smoking in pregnancy. Where multiple SNPs were associated with individual CpG loci, principle component analysis was performed to identify principle components that explained ≥70% of the proportion of variance (Table S3 in the Data Supplement).

### Network and Gene Ontology Enrichment

Protein-protein interaction networks (PPI) were examined using the Search Tool for the Retrieval of Interacting Genes/Proteins.^46^ Genes annotated to dmCpGs associated with outcomes (FDR adjusted *P* value ≤0.25) were entered into Search Tool for the Retrieval of Interacting Genes/Proteins and visualized in Cytoscape.^47^ The properties of the PPI network were calculated under default parameters, and only connected nodes that generated a network with a PPI enrichment *P*≤0.05 were retained for subsequent analysis. Large networks were further segmented using the MCODE algorithm^48^ in Cytoscape using default parameters. Enriched gene ontology terms were determined using BiNGO^49^ to examine over-represented gene ontology terms.

## Results

### Study Participants

DNA was extracted from cord blood samples from SWS infants who had measures of cardiovascular structure or function at a median age of 9.2 years. Table 1 shows characteristics of the 470 participants for which DNA methylation data was available; Table S1 provides a comparison of SWS participants with and without methylation data.

**Table 1.**
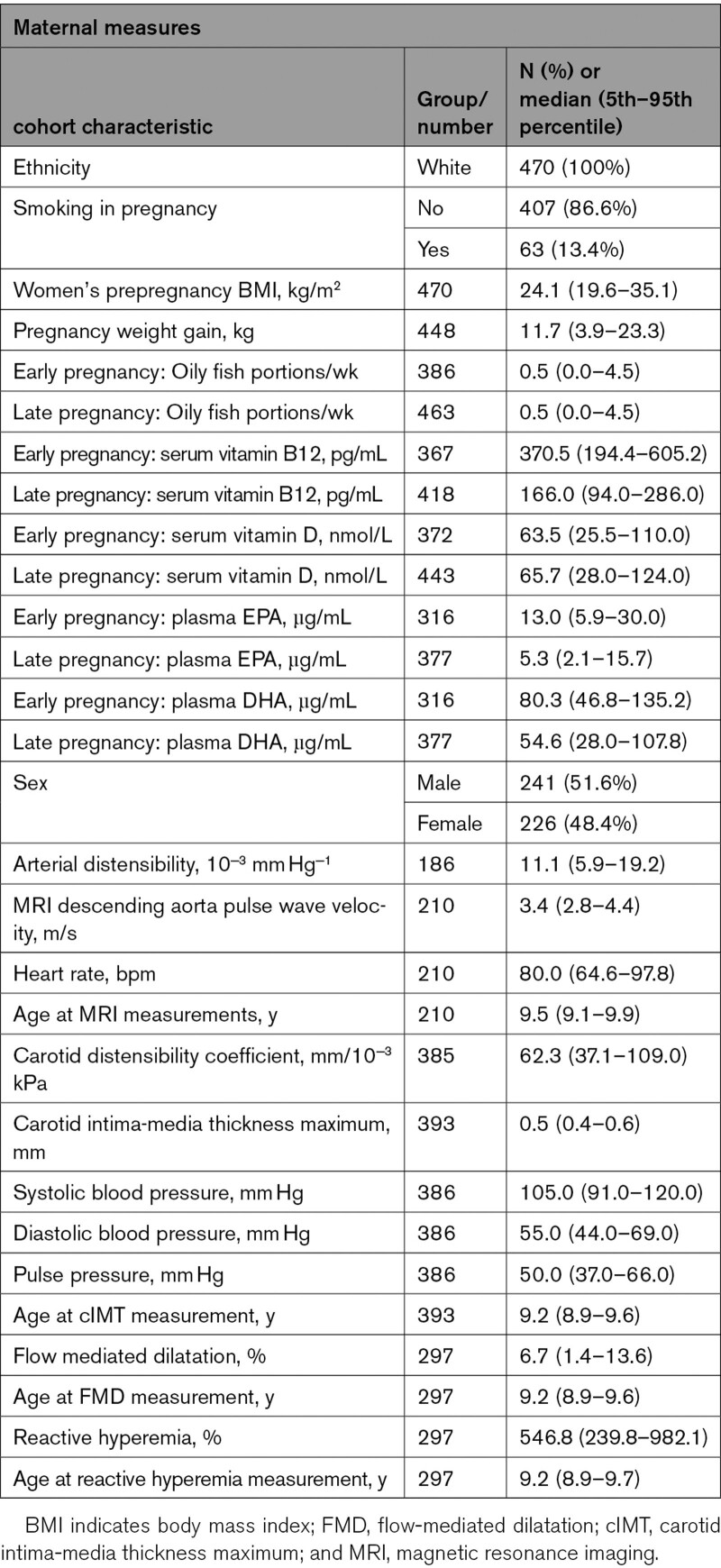
Cohort Characteristics

### Relationship Between Cord Blood DNA Methylation and aPWV at 8 to 9 Years

**Figure 1.**
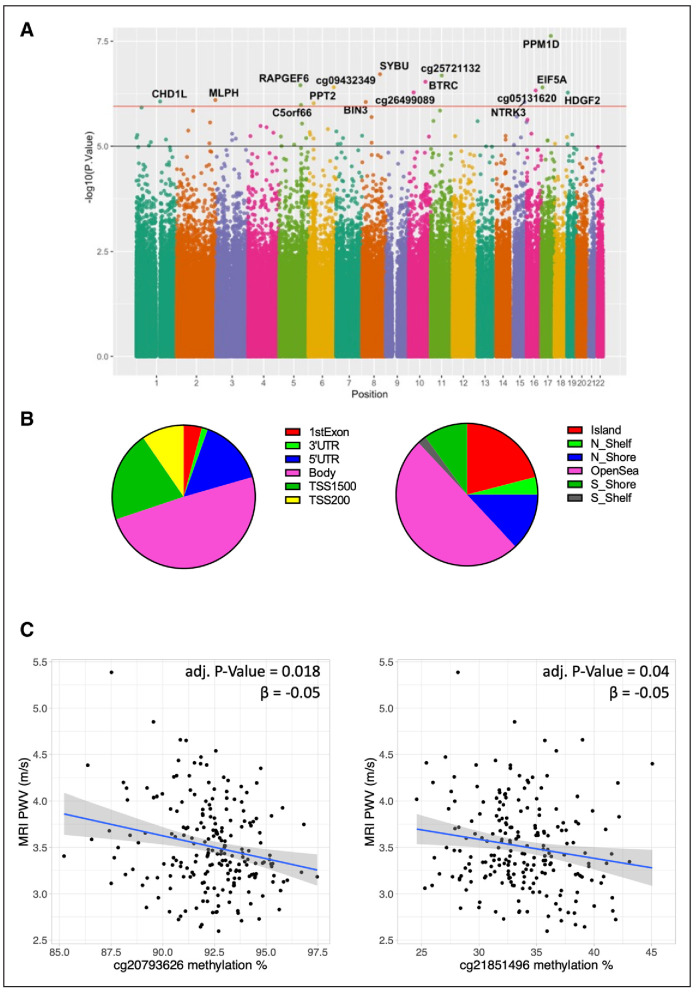
**Associations between cord blood DNA methylation and child magnetic resonance imaging (MRI) pulse wave velocity (PWV).A**, Manhattan plot showing genome-wide distribution of dmCpGs. **B**, Enrichment for genomic features (false discovery rate ≤0.25) relative to gene group and to CpG density. **C**, Associations between the top associated dmCpGs (%) and 8–9 y MRI PWV (m/s).

Cord blood DNA methylation was analyzed in relation to the measures of arterial stiffness and structure at 8 to 9 years of age. There were 16 differentially methylated CpG dinucleotides (dmCpGs) with an FDR of ≤0.05 associated with aPWV at 8 to 9 years (Figure 1A; Table 2). The most significantly associated dmCpGs were cg20793626 (Figure 1C), located within the gene body of protein phosphatase, Mg2+/Mn2+ dependent 1D (*PPM1D*), and cg21851496, located within the north shore of syntabulin (*SYBU*). The other identified dmCpGs were primarily located within open seas or, when associated with a gene, within CpG islands (Figure 1B). A 1% decrease in methylation of cg20793626 was associated with a 0.06 m/s increase in aPWV, while the largest effect size observed was with methylation of cg19669439 within the promoter of β-transducin repeat containing E3 ubiquitin protein ligase (*BTRC*), where an increase of 1% methylation was associated with an increase in aPWV of 0.24 m/s (Table 3). All associations with aPWV remained significant after adjustment for child’s height, BMI at 8 to 9 years, maternal age at birth, maternal educational attainment, and parity (Table S2A). Analysis was also performed to detect differentially methylated regions^50^; however, no differentially methylated regions were detected (data not shown).

**Table 2.**
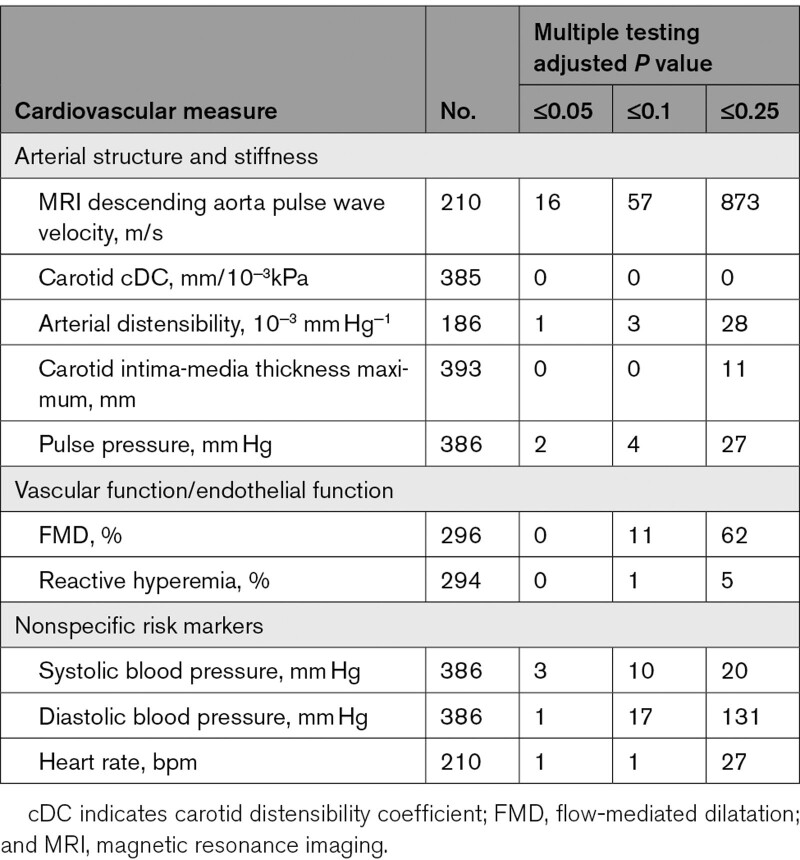
Summary Table of Epigenome Wide Association Study Analysis

**Table 3.**
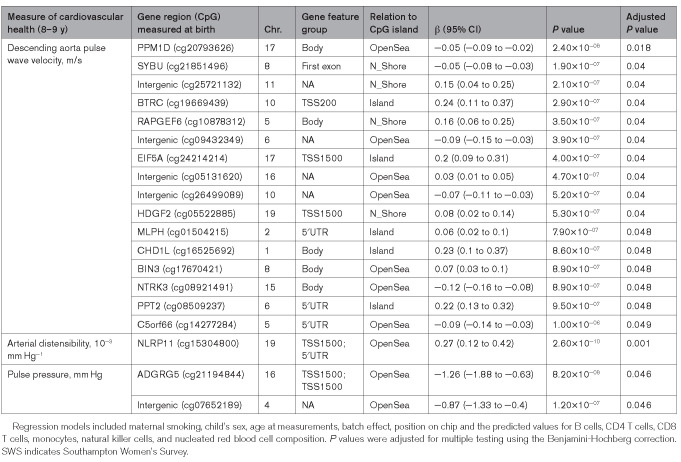
Significant Associations Between Cord Blood Methylation and Measures of Cardiovascular Structure and Stiffness at Age 8–9 y in SWS Children

### Enrichment of aPWV-Associated dmCpGs in Pathways Associated With DNA Repair, Metabolic Process, and Protein Transport

To gain a better understanding of the functional significance of the methylation changes, genes associated with the aPWV-associated dmCpGs at an FDR ≤0.25 were entered into Search Tool for the Retrieval of Interacting Genes/Proteins to generate a PPI network (Figure S2). Of the 874 CpGs associated with aPWV (FDR ≤0.25), 628 were associated with a unique gene. The resulting PPI network (FDR ≤0.05) had 540 nodes and 1011 interactions. To determine key modules in the PPI network, the network was subdivided into clusters using the MCODE algorithm, resulting in 12 individual modules being identified. The significant gene ontology terms associated with these clusters were DNA repair (FDR, 9.03×10^−11^), cellular macromolecular metabolic process (FDR, 4.31×10^−07^), endoplasmic reticulum to Golgi mediated transport (FDR, 1.87×10^−07^), positive regulation of catalytic activity (FDR, 1.07×10^−05^), and carbohydrate metabolic process (FDR, 2.51×10^−03^). There were limited PPIs related to dmCpGs associated with arterial distensibility or the carotid distensibility coefficient, with no enrichment of pathways.

### A Combined Model for DNA Methylation Signature at Birth Predictive of aPWV at 8 to 9 Years

Multivariate linear regression was used to assess the variance in aPWV explained by the dmCpGs which independently associated with aPWV. A combined model using the methylation status of 14 of the PWV dmCpGs explained 32.4% of the total variance; after adjustment for batch effect, position on array and cell type correction these 14 dmCpGs explained 42.8% of the total variance in PWV. Additionally correcting for maternal smoking, child’s sex and age at measurement the model explained 45.4% of the total variance in late childhood aPWV (Table S8).

### Relationship Between Cord Blood DNA Methylation and Further Measures of Arterial Stiffness and Structure at 8 to 9 Years

To assess whether cord blood DNA methylation signatures were also associated with further measures of arterial structure, we analyzed methylation at birth in relation to arterial distensibility, pulse pressure, carotid distensibility coefficient, and carotid intima-media thickness. There was one dmCpG associated with arterial distensibility (measured in 186 children); this was cg15304800 (Table 3) located within the 5′ promoter region of the NOD-like receptor family pyrin domain containing 11 (*NLRP11*) gene. Two dmCpGs, cg21194844 (*ADGRG5*) and cg07652189 (intergenic), were associated with pulse pressure. There were no dmCpGs with an FDR of ≤0.05 that showed a significant association with carotid distensibility coefficient (n=389) or carotid intima-media thickness (n=393). CpG sites associated with arterial distensibility and pulse pressure (FDR of ≤0.25) were compared, but there was no overlap with dmCpGs associated with aPWV (Figure S1).

### Associations Between Cord Blood DNA Methylation and Measures of Endothelial Function

FMD and RH assess conduit artery endothelial function as well as small vessel physiology, reflecting different aspects of vascular biology. We, therefore, examined the associations of DNA methylation at birth with FMD and RH at 8 to 9 years. There were no dmCpGs associated with FMD or RH at an FDR of 0.05, but 11 FMD- and 1 RH- associated dmCpGs at an FDR of ≤0.1. The association between DNA methylation and systolic blood pressure and diastolic blood pressure was also examined. There were 3 dmCpGs associated with systolic blood pressure (FDR of ≤0.05; Table 4): cg14407341 within the chimerin 2 gene (*CHN2*), cg05902531 within carboxy-terminal domain, RNA polymerase II, polypeptide A (*CTDSP2*) gene and cg12256233 within an open sea region, and 1 dmCpG associated with diastolic blood pressure—cg24745895, also within an open sea region.

**Table 4.**
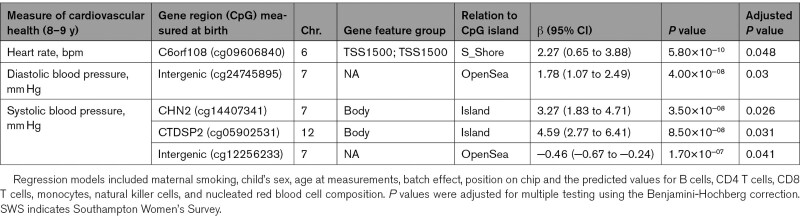
Significant Associations Between Cord Blood Methylation and General Measures of Cardiovascular Health at Age 8–9 y in SWS Children

### Associations Between Maternal Lifestyle Factors and dmCpGs Associated With aPWV

**Figure 2.**
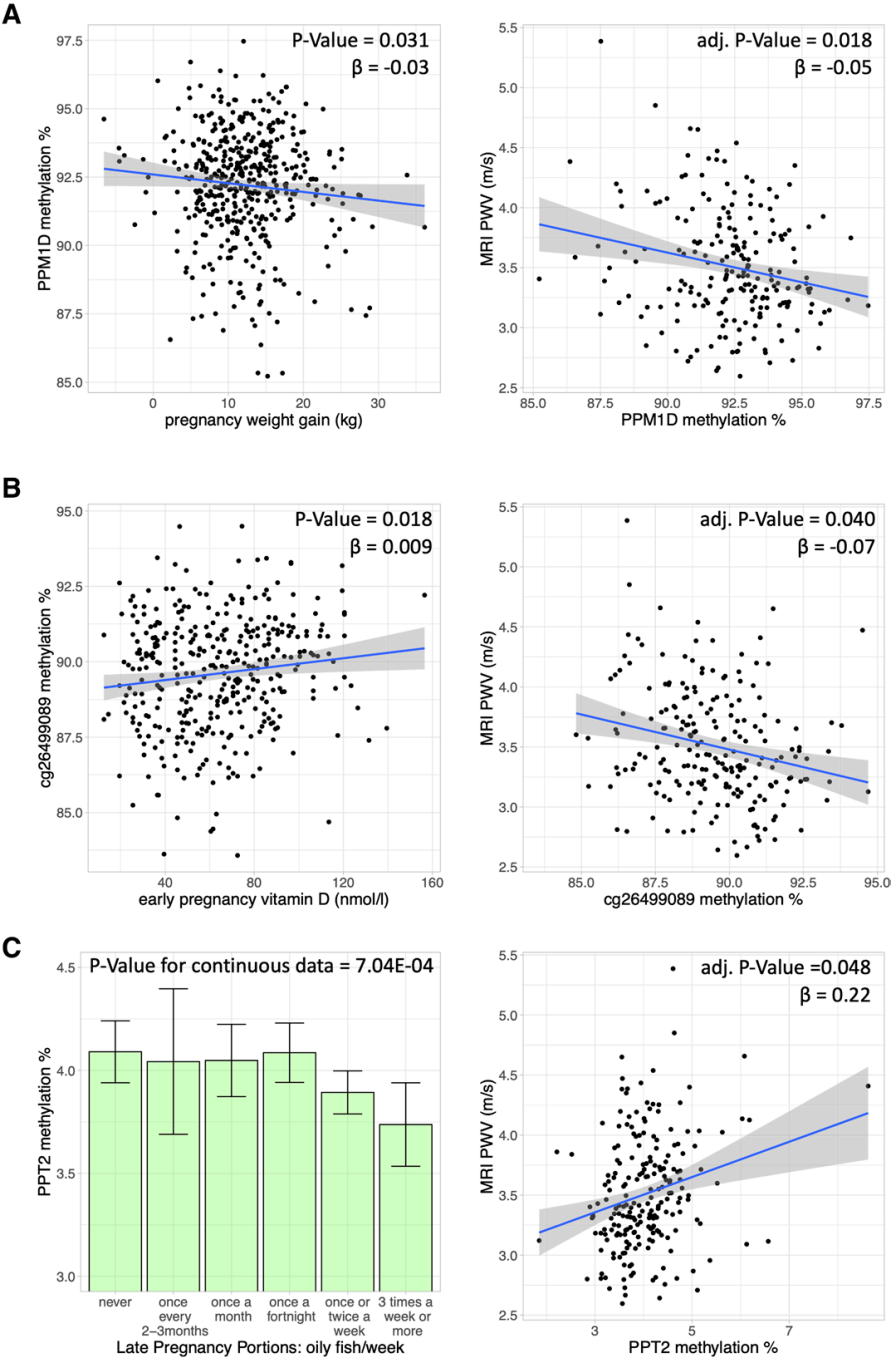
**Associations between maternal factors and dmCpG methylation.** Associations between (**A**) pregnancy weight gain, PPM1D dmCpG methylation, and later associations with magnetic resonance imaging (MRI) pulse wave velocity (PWV); (**B**) early pregnancy circulating vitamin D levels, cg26499089 dmCpG methylation, and later associations with MRI PWV; and (**C**) late pregnancy maternal oily fish consumption compared with PPT2 (palmitoyl-protein thioesterase 2) dmCpG methylation, and later associations with MRI PWV.

Maternal BMI, weight gain in pregnancy, and maternal smoking have all been linked with CVD risk in the offspring.^17,22^ We, therefore, investigated the relationship between these factors and the methylation status of the CpGs loci associated with aPWV. Five of the 16 aPWV-dmCpGs were associated with a number of these modifiable risk factors (Table 5). Cg05522885, located within *HDGF2*, was positively associated with prepregnancy BMI (β=0.03 [95% CI, 0.005–0.06], *P*=0.02), cg24214214 (*EIF5A*) positively associated with weight gain during pregnancy (β=0.02 [0.005–0.03], *P*=0.004), while cg20793626 (*PPM1D*; β=−0.03 [−0.06 to −0.003], *P*=0.03) and cg09432349 (β=−0.03 [−0.06 to −0.01], *P*=0.005) inversely associated with pregnancy weight gain (Figure 2A). Methylation of cg25721132 (intergenic; β=0.27 [0.07–0.47], *P*=0.008) was positively associated with maternal smoking during pregnancy. There were no associations between maternal age at birth, educational attainment or parity, and aPWV-associated dmCpG sites (data not shown).

**Table 5.**
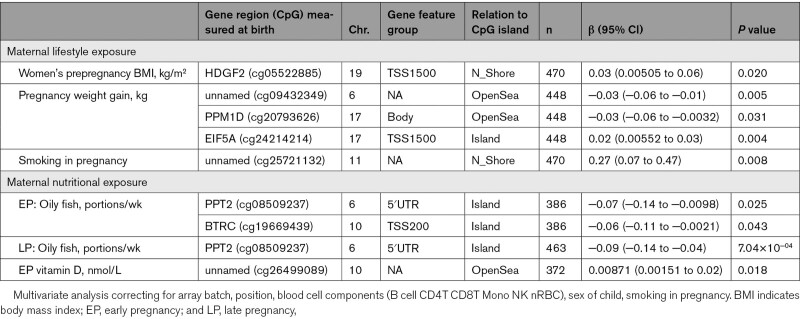
Associations Between Maternal Factors and dmCpG Sites Associated With Aortic PWV

### Associations Between Maternal Dietary Factors and dmCpGs Associated With aPWV

Maternal dietary intake has been associated with CVD risk in the offspring,^18–20^ with components such as vitamin D and oily fish^16,17^ suggested to play a role in establishing the offspring’s cardiovascular health. We investigated the relationships of maternal serum vitamin D, vitamin B12, energy intake (kcal/d), and oily fish consumption with the aPWV-associated dmCpGs. There was a positive association between serum vitamin D concentration in early pregnancy and the methylation of cg26499089 (intergenic; β=0.009 [0.0015–0.02], *P*=0.018; Table 5, Figure 2B), but no associations between the aPWV-associated dmCpGs and serum vitamin B12 concentrations. Late pregnancy maternal oily fish consumption, analyzed as a continuous variable, was inversely correlated with methylation of cg19669439 (*BTRC*) in early pregnancy (β=−0.06 [−0.11 to −0.002], *P*=0.043), and oily fish intake in both early and late pregnancy with methylation of cg08509237, located within the first exon of *PPT2* (palmitoyl-protein thioesterase 2), β=−0.07 (−0.14 to −0.0098), *P*=0.025 and β=−0.09 (−0.14 to −0.04), *P*=0.0007, respectively (Figure 2C). EPA and DHA concentrations measured in blood (μg/mL) in early and late pregnancy were analyzed in relation to methylation of CpG sites associated with aPWV at 8 to 9 years. There was an inverse relationship between plasma phosphatidylcholine DHA, but not EPA, concentration and the methylation status of PPT2 (cg08509237; Table S3). Interaction terms were examined for identified maternal factors and CpG methylation (adjusting for the main effects of both the maternal factor and CpG methylation), alongside covariates (Table S4). The interaction term for early pregnancy oily fish consumption with PPT2 (cg08509237) methylation was significant at the 5% level (β=0.075 [0.002–0.15], *P*=0.045). Examination of the relationship between cg08509237 methylation and aPWV in groupings based upon early pregnancy oily fish consumption was consistent with a threshold effect, where methylation had a stronger association with aPWV for individuals in the group with the lowest consumption of oily fish (once every 2 to 3 months or less) in early pregnancy (Figure S3). The interaction term for smoking in pregnancy with cg25721132 (intergenic) methylation had a larger effect size of β=0.184 (95% CI, −0.03 to 0.4) but did not reach significance (*P*=0.089).

### Influence of Genetic Variation on dmCpGs Associated With aPWV

To investigate the influence of genetic variation on the 873 aPWV-dmCpGs (FDR ≤0.25) a genome-wide screen^45^ was performed to identify methylation quantitative trait loci; 148 of the dmCpG sites had significant associations with 556 genetic variants; this included methylation quantitative trait loci associated with 5 of the 16 most significant (FDR ≤0.05) aPWV-associated dmCpGs (Table S5). Genetic influence at *PPM1D*, *EIF5A*, *NTRK3*, and *C5orf66* was modest, with multivariate models accounting for between 3% and 14.6% of the variance in CpG methylation at different sites (Table S6). These genetic variants showed no independent associations with aPWV (Table S7A), and associations between methylation of *PPM1D*, *EIF5A*, *NTRK3*, and *C5orf66* and aPWV remained significant when accounting for these genetic variants (Table S7B). Methylation at the aPWV-associated intergenic CpG site cg05131620 was strongly influenced by genotype; methylation quantitative trait loci at 16 loci across a 44Kb region (Chr16:55460729-55504521) associated in cis with methylation of cg05131620 (Chr16:55478717). A model including genotype and covariates explained 77.6% of the total variance in cg05131620 methylation, while a model of the covariates alone explained 35%. Genetic variation associated with cg05131620 was directly associated with aPWV (Table S7A). Accounting for genotype weakened the significance of the association of cg05131620 with aPWV (β=0.03 [−0.001 to 0.06], *P*=0.052 compared with β=0.03 (0.01 to 0.04), *P*=7.92×10^−04^ without genotype in matched samples, Table S7B). The potential role of an interaction between genotype and methylation on aPWV levels was also examined, but no interaction terms were significant (Table S7C).

## Discussion

There is increasing evidence that an adverse early life environment is associated with increased risk of CVD in later life.^22^ The mechanisms by which the maternal environment can induce such long-term effects on the offspring have been suggested to involve the altered epigenetic regulation of genes.^21^ Here, we found that DNA methylation signatures at birth were associated with aPWV at age 8 to 9 years, and that a number of modifiable maternal risk factors, particularly aspects of diet and weight gain in pregnancy associated with methylation of these CpGs sites. The identification of epigenetic markers of future CVD risk together with modifiable risk factors that influence the methylation status of such markers may allow the early stratification of individuals at increased risk of CVD and potentially facilitate development of preventive strategies to reduce the offspring’s later risk of CVD.

Increased arterial stiffness is one of the earliest signs of pathogenic changes within the structure of the vessel wall.^9^ Here, we found 16 dmCpG sites which were related to aPWV. The dmCpG most significantly associated with aPWV was located within *PPMID*, which encodes a serine/threonine phosphatase that dephosphorylates proteins involved in the DNA damage response. Both animal and human studies suggest that impairment to the DNA damage response and repair pathways is linked to increased arterial stiffness and elevated BP.^51,52^ Here, we found that higher *PPMID* gene body methylation was associated with decreased aPWV. As gene body DNA methylation is generally associated positively with transcription, this would suggest that, in line with previous findings, higher expression of *PPMID* and a suppression of the DNA damage response is associated with increased arterial stiffness. Moreover, pathway analysis revealed that DNA repair was the most significantly enriched pathway among the dmCpGs that were associated with aPWV, including DNA repair genes *RAD23A*, *RAD51B*, *CHD1L*, *REV1*, and *XPA*.

An increase in aPWV of 1 m/s has been associated with a 15% increase in relative risk of cardiovascular-related mortality.^53^ In our data, cg20793626 (*PPM1D*), the dmCpG most strongly associated aPWV, was associated with an increase in aPWV of 0.06 m/s, which equates to an increase in cardiovascular-related mortality risk of 0.9% per 1% decrease in DNA methylation. Given that *PPM1D* DNA methylation has a range of 12% within our cohort, this potentially represents a substantial variation in risk across the cohort. Moreover, combining cg20793626 in a multivariate model with 13 further aPWV-associated dmCpGs alongside covariates explained 45.4% of the total variance observed in aPWV, suggesting that the methylation signature associated with aPWV may have utility as a predictor of future cardiovascular health.

Several of the dmCpGs in the multivariate model have robust CVD links; cg17670421 is located within the *BIN3* gene, which has been reported to play a role in cardiomyocyte homeostasis and is downregulated in cardiomyopathy, while plasma *BIN1* levels have been shown to predict heart failure.^54^ Additionally, methylation within *MLPH* was found to have a strong predictive relationship with the incidence of coronary heart disease in a meta-analysis of 11 461 adults. The CpG site in *MLPH* we identified in our study was not present on the array used by Agha et al^55^; however, the discovery that further CpG sites within this gene are linked to PWV does support methylation status of the *MLPH* gene as a marker of CVD risk.

The environment, particularly the early life environment, has also been shown to influence DNA methylation as well as the phenotype and is a key determinant of an individual’s risk of developing CVD in adult life.^56^ Exposure to maternal obesity is strongly linked to death from coronary heart disease, stroke, and other cardiovascular events in the adult offspring,^57,58^ while maternal weight gain in pregnancy is associated with increased left ventricular mass in the child at birth.^59^ Of the 16 dmCpG sites that were associated with aPWV, one was also associated with prepregnancy BMI and 3 were associated with weight gain during pregnancy, suggesting an influence of maternal body composition both before and during pregnancy. Maternal BMI and excessive weight gain during pregnancy are major drivers of childhood adiposity,^60,61^ which has also been linked with later CVD risk.^62^ However, adjusting for the child’s BMI did not attenuate the associations between DNA methylation and aPWV in our analyses. One interpretation is that the apparent influence of maternal BMI or weight gain during pregnancy on these DNA methylation signatures may involve pathways that are independent of the child’s BMI.

Exposure to maternal smoking during pregnancy has been associated with reduction in arterial elasticity and elevated systolic blood pressure in children.^63,64^ The present findings show that maternal smoking during pregnancy was associated with increased methylation of cg25721132, an intergenic dmCpG for which increased methylation was associated with greater aPWV. This suggests that maternal smoking may influence arterial stiffness at least in part through epigenetic processes.

A number of studies have reported associations between maternal diet and later CVD risk in the offspring: for example, we previously showed that increased oily fish consumption in late pregnancy was associated with a reduction in the child’s PWV,^19^ while other studies have shown that dietary supplementation with omega-3 polyunsaturated fatty acid in adults reduces the incidence of cardiovascular events, as well as lowering BP and heart rate.^65,66^ *PPT2* encodes palmitoyl-protein thioesterase-2 involved in fatty acid elongation. Here, we found that higher methylation of cg08509237 located within the 5′UTR of *PPT2*, for which methylation status was related to aPWV, was associated with decreased intake of oily fish in early and late pregnancy. Consistent with this, a lower DHA concentration in late pregnancy, for which oily fish is the major dietary source,^15^ was also associated with increased *PPT2* methylation. The association of cg08509237 (PPT2) methylation at birth with later aPWV varied according to consumption of oily fish in early pregnancy, with further analysis indicating that consumption above a threshold of one or more portions per month during early pregnancy weakened the association between methylation and increased aPWV. This threshold effect was observed in relation to early, but not late, pregnancy—suggesting that early pregnancy is a critical developmental window where diet may influence the way in which DNA methylation relates to later aPWV. Interestingly, genetic variation within *PPT2* has previously been linked to docosapentaenoic acid (22:5n-3) levels in a trans-ethnic meta-analysis that suggested a role for *PPT2* in polyunsaturated fatty acid metabolism.^67^ Oily fish consumption in early pregnancy was also associated with the methylation status of cg19669439, which is located within the promoter region of β-transducin repeat containing E3 ubiquitin protein ligase (*BTRC*), one of the top dmCpG sites associated with later aPWV. BTRC (also known as β-TrcP1) mediates ubiquitination and degradation of β-catenin, which plays an essential role in heart development as well as cardiac tissue homoeostasis in adults.^68^ Moreover, reduced DNA methylation and increased gene expression of BTRC within peripheral blood has been reported in patients with coronary artery disease.^69^ Thus, these findings suggest a link between maternal polyunsaturated fatty acid intake, altered epigenetic regulation of cardiovascular development in the fetus, and childhood measures of CVD risk. Members of the PPAR family of fatty acid-binding transcription factors interact with both DNA methyl transferases^70^ and demethylases,^71^ while supplementation with fatty acids has been directly linked to changes in DNA methylation.^72^

Fixed genetic variation can also influence DNA methylation patterns,^45^ and we identified methQTLs associated with 5 of the 16 aPWV-associated CpG sites. Taken together our results show that both prenatal modifiable CVD risk factors and fixed genetic variants can influence the PWV-associated methylation signature in infants; however, after adjusting our model to account for these factors, associations between methylation at birth and later cardiovascular measures generally remained significant. Identifying additional factors that modulate the methylation status at these sites, together with a better understanding of whether such epigenetic changes play a causal role in the development of increased arterial stiffness, or are simply markers of this process, will allow us to further refine such signatures and facilitate their utility as prognostic tools.

There are several strengths of this study. It is the first epigenome wide association study to identify DNA methylation changes at birth that are associated with extensive measures of both cardiovascular structure and function in late childhood, permitting the detection of early epigenetic markers associated with cardiovascular risk in children. Prior studies that have examined methylation changes in relation to CVD risk have focussed upon mid- to late-adulthood methylation changes and associations with concurrent CVD, when damage to vessel walls is more pronounced. Second, aPWV was measured in this study by magnetic resonance imaging allowing for a true measurement of vascular lumen length (distance between the flow acquisitions)—this is in contrast to other PWV techniques that rely upon an approximated value from a body surface measurement, which has implications for the accuracy of the derived velocity.^3,73^ Additionally, we used the Illumina 850K array, which has greater coverage of CpG sites in important regulatory regions of the genome associated with cardiovascular risk than previous genome-wide studies that used the Illumina 27K or 450K arrays. For example, the strongest associations we observed were from probes unique to the Illumina 850K array, and almost half of our significant associations are not represented on the 450K platform. In our combined model, 6 of the 14 CpG sites are not present on the 450K platform (cg20793626, cg21851496, cg09432349, cg05131620, cg01504215, cg17670421), including *PPM1D*—the strongest association with aPWV. Whether these epigenetic markers are causally involved in mediating the changes in arterial stiffness is not known. Nonetheless, as markers of early vascular pathophysiology they may be useful in early identification of individuals with an increased CVD risk during the lifecourse and assist in the evaluation of potential interventions to reduce this risk.

Our study has several limitations. First, DNA methylation was examined in cord blood and, although we have corrected our analysis for cord blood cell composition, the longer-term methylation status and functional significance of these changes is unknown. DNA methylation changes in nucleated blood cells may reflect changes in immune function, or be related to inflammation, so further work is needed to elucidate the mechanistic links between the dmCpG sites we identified and alterations in an individual’s cardiovascular health. Furthermore, our observations represent a child’s cardiovascular phenotype at a single time point at age 8 to 9 years, rather than a longitudinal assessment of cardiovascular health. The children we studied were all of White ethnicity and extension to and replication in further cohorts is necessary to validate and determine the generalizability of these observations. Additionally, we were not able to examine gene expression in cord blood in relation to these DNA methylation changes, nor was data available on maternal DNA methylation patterns to examine epigenetic regulation of marks identified here in relation to the maternal methylome.

### Conclusions

To our knowledge, this is the first epigenome wide association study to investigate the relationship between perinatal DNA methylation with markers of cardiovascular structure and function in childhood. The findings showed that the methylation status of individual CpG loci in umbilical cord blood at birth were predictive of arterial stiffness in children at age 8 to 9 years, with a multivariate model containing 14 dmCpGs as well maternal and array covariates including cell type correction explained 45.4% of the variance in the child’s aPWV. Furthermore, several of the aPWV-associated dmCpGs were also associated with a range of modifiable risk factors, including maternal oily fish intake, suggesting that methylation signatures at birth predictive of later CVD risk are influenced by maternal health, diet, and lifestyle choices. The identification of such modifiable lifestyle factors before pregnancy, and in both early as well as late pregnancy, may permit the development of new intervention strategies to improve the cardiovascular health of the offspring.

### Perspectives

DNA methylation patterns at birth have been related to health outcomes in later life, potentially reflecting exposures in utero and thus aspects of the maternal environment. We conducted the first epigenome-wide association study of DNA methylation signatures at birth related to clinical measures of cardiovascular structure and function in children aged 8 to 9 years. DNA methylation patterns at birth were related to child’s BP, pulse pressure, arterial distensibility, and aPWV; early markers of cardiovascular risk. Maternal influences modified these methylation patterns, in particular, oily fish consumption during pregnancy, with an interaction between early pregnancy oily fish consumption and later childhood pulse wave velocity. Taken together, this work identifies predictive biomarkers of later CVD risk in children and demonstrates the potential for maternal factors such as diet in pregnancy to modify these markers and promote cardiovascular health in the next generation.

## Acknowledgments

R. Murray performed the molecular laboratory work. R. Murray and K.A. Lillycrop drafted the article. R. Murray, N. Kitaba, E. Antoun, and P. Titcombe performed the statistical analysis and prepared the tables/figures. S.J. Barton participated in the analysis. S.J. Barton, C. Cooper, H.M. Inskip, G.C. Burdge, P.A. Mahon, J. Deanfield, J. Bryant, C. Peebles, J.P. Halcox, E.A. Ellins, K.A. Lillycrop, K.M. Godfrey, and M.A. Hanson participated in the study design and collected the samples/physiological measurements. K.A. Lillycrop, K.M. Godfrey, and M.A. Hanson conceived of the study, its design, and its coordination. All authors helped draft the article, participated in article editing, and read/approved the final article. We wish to acknowledge the contributions of Helena Fisk and Philip Calder for performing the analysis of plasma fatty acid composition from maternal samples in early and late pregnancy.

## Sources of Funding

This work was funded by the British Heart Foundation (RG/15/17/31749). The Southampton Women’s Survey has received funding from the Medical Research Council, Dunhill Medical Trust, British Heart Foundation, Arthritis Research UK, Food Standards Agency, National Institute for Health Research (NIHR) Southampton Biomedical Research Centre, and the European Union’s Seventh Framework Programme (FP7/2007–2013), project EarlyNutrition, under grant agreement 289346 and the European Union’s Horizon 2020 research and innovation programme (LIFECYCLE, grant agreement No. 733206). K.M. Godfrey is supported by the UK Medical Research Council (MC_UU_12011/4), the National Institute for Health Research (NIHR Senior Investigator [NF-SI-0515-10042], NIHR Southampton 1000DaysPlus Global Nutrition Research Group [17/63/154] and NIHR Southampton Biomedical Research Centre [IS-BRC-1215-20004]), and the European Union (Erasmus+ Programme ImpENSA 598488-EPP-1-2018-1-DE-EPPKA2-CBHE-JP).

## Disclosures

K.M. Godfrey and G.C. Burdge have received reimbursement for speaking at conferences sponsored by companies selling nutritional products. K.A. Lillycrop and K.M. Godfrey are part of academic research programs that have received research funding from Abbott Nutrition, Nestec, Danone and BenevolentAI Bio Ltd. G.C. Burdge has received research funding from Abbott Nutrition, Nestec, and Danone and has been a scientific advisor to BASF. J. Deanfield is on the speakers’ bureaus for Pfizer, Sanofi-Aventis, and AstraZeneca. J.P. Halcox is on the speakers’ bureaus for Pfizer, Merck Sharpe & Dohme, and Sanofi-Aventis. The other authors report no conflicts.

## Supplementary Material



## Novelty and Significance

### What Is New?

- Identified at birth 16 novel CpG sites with methylation levels associated with aortic pulse wave velocity, an early measure of arterial stiffness linked to elevated systolic blood pressure and increased cardiac afterload.
- Developed a multivariate model that combines differential methylation at 14 CpG sites which, with other covariates, explained 45.4% of the variance in late childhood pulse wave velocity, potentially providing a strong predictive tool of future risk.
- Found evidence that maternal weight gain and nutrition during pregnancy influenced these early epigenetic biomarkers of cardiovascular risk.

### What Is Relevant?

- Epigenome-wide association study examining methylation levels at birth finding associations with later measures of systolic and diastolic blood pressure, as well as pulse pressure.
- First study to use magnetic resonance imaging-derived measures of pulse wave velocity to identify epigenetic biomarkers of arterial stiffness, a driver of increased systolic blood pressure and cardiac afterload in later life, as well as an independent risk factor for cardiovascular disease.
- Study examined both fixed and modifiable factors that could influence these epigenetic marks, identifying an underlying interaction of maternal diet with methylation and later pulse wave velocity.

### Summary

Analysis of DNA methylation levels measured at birth in a highly characterized cohort with in-depth measures of cardiovascular structure and function in late childhood identified epigenetic marks associated with pulse wave velocity, blood pressure, pulse pressure, arterial distensibility, and heart rate. Examination of both fixed and modifiable prenatal factors identified a relationship between components of the maternal diet, DNA methylation, and later cardiovascular health.
