# Supplementary figures and images for: Influence of Maternal Lifestyle and Diet on Perinatal DNA Methylation Signatures Associated With Childhood Arterial Stiffness at 8 to 9 Years
Source: Hypertension. 2021 Jul 19;78(3):787–800. doi: 10.1161/HYPERTENSIONAHA.121.17396 (PMC8357051; doi:10.1161/HYPERTENSIONAHA.121.17396)

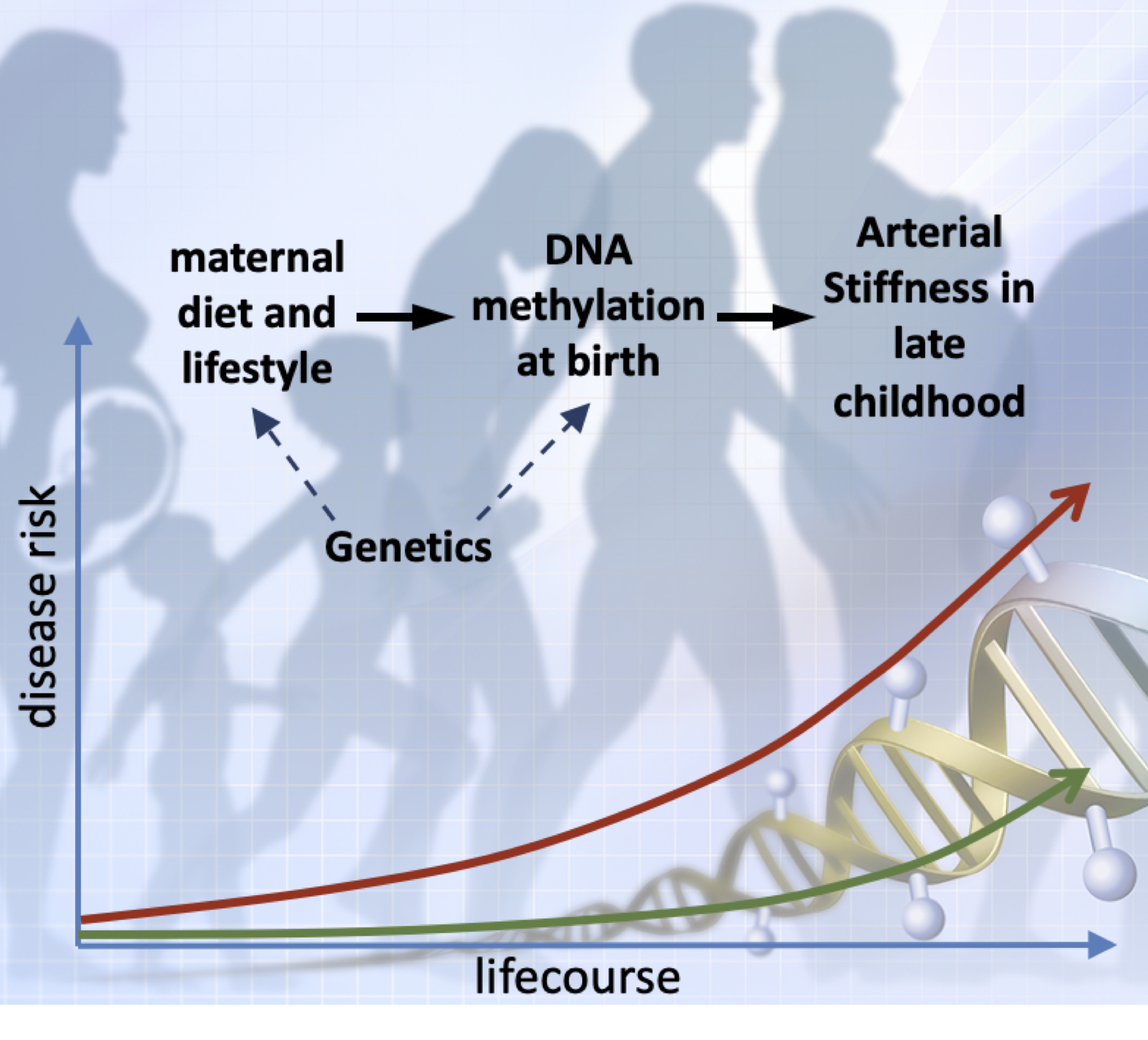

Supplement: Supplementary file 1 [file hyp-78-787-s001.jpg]
