# Supplementary material for: Influence of Maternal Lifestyle and Diet on Perinatal DNA Methylation Signatures Associated With Childhood Arterial Stiffness at 8 to 9 Years
Source: Hypertension. 2021 Jul 19;78(3):787–800. doi: 10.1161/HYPERTENSIONAHA.121.17396 (PMC8357051; doi:10.1161/HYPERTENSIONAHA.121.17396)
Supplement: Supplementary file 2 [file hyp-78-787-s002.docx]

DATA SUPPLEMENT

**Influence of maternal lifestyle and diet on perinatal DNA methylation signatures associated with childhood arterial stiffness at 8-9 years**

Robert Murray^1^

Negusse Kitaba^1^

Elie Antoun^1,4^

Philip Titcombe^2^

Sheila Barton^2^

Cyrus Cooper^2^

Hazel M. Inskip^2,3^

Graham C Burdge^1^

Pamela A. Mahon^2^

John Deanfield^8^

Julian P. Halcox^5^

Elizabeth A. Ellins^5^

Jennifer Bryant^6^

Charles Peebles^7^

Karen Lillycrop^3,4*^

Keith M. Godfrey^1,2,3*^

Mark A. Hanson^1,3*†^

EpiGen Consortium

^1^ School of Human Development and Health, Institute of Developmental Sciences Building, Faculty of Medicine, University of Southampton, Southampton, UK

^2^MRC Lifecourse Epidemiology Unit, University of Southampton, Southampton, UK

^3^NIHR Southampton Biomedical Research Centre, University of Southampton and University Hospital Southampton NHS Foundation Trust, Southampton, UK

^4^Centre for Biological Sciences, Faculty of Natural and Environmental Sciences, University of Southampton, Southampton, UK

^5^Swansea University Medical School, Swansea University, Swansea, UK

^6^Department of Cardiac Magnetic Resonance Imaging, National Heart Centre Singapore, Singapore

^7^Wessex Cardiothoracic Centre, Southampton University Hospitals NHS Trust, Tremona Road, Southampton SO16 6YD, UK

^8^Institute of Cardiovascular Sciences, University College London, London, UK.

*Joint senior authors

^†^ Corresponding author

**DATA SUPPLEMENT – Methods**

***Measures of maternal diet and lifestyle in the SWS***

In the SWS, assessments of lifestyle, diet (by validated food frequency questionnaire^31^) and anthropometry were performed at study entry and then in early (11-week) and late (34-week) gestation in those women who became pregnant. Smoking status in pregnancy was ascertained at the 11- and 34-week interviews. Pre-pregnancy data on the analysis sample were collected at a median of 1.1 year before conception. Because women’s weight tended to increase with age (by ’0.4% each year), a regression adjustment was used to remove the average increases in weight between data collection and conception, so that all women’s weights before pregnancy were adjusted to their predicted weight on the day of conception. Pregnancy weight gain was calculated as the difference between pre-pregnancy weight and weight at 34-weeks’ gestation. At the time of the 11 week and 34 week scans, a research nurse obtained venous blood samples, and maternal serum was frozen at -80°C for analysis. 25-hydroxyvitamin D concentrations were analysed by radioimmunoassay (Diasorin, Stillwater, Minnesota, USA). This assay measures both vitamin D2 and D3. The assay met the requirements of the UK National External Quality Assurance Scheme (NEQAS) and intra-assay and inter-assay coefficients of variation were less than 10%.

***Blood pressure and carotid intima-media thickness***

Systolic and diastolic BP and pulse rate of the children were measured using a Dinamap Critikon 8100 monitor. Carotid IMT and distensibility were assessed using a Philips iE33 dedicated vascular ultrasound system (Philips Healthcare, Guilford, UK). Measurements were made once after a 10-15 minute rest, with the child supine and the head turned 45 degrees to the right of the midline. The left carotid artery was imaged longitudinally with a dedicated vascular linear transducer (3-11MHz power), just proximal to the carotid bifurcation, demonstrating clearly defined intima on both anterior and posterior walls. A video-captured sequence which included at least 4 cardiac cycles was acquired over 10 seconds. Immediately after acquiring an optimum trace, the BP cuff (upper arm, on the same side) was inflated and systolic and diastolic BP and pulse rate were recorded in mmHg and beats/min respectively. To calculate carotid IMT and distensibility, images were analysed using MIA (MIA, IA, USA) software. Maximum cIMT was measured as the maximum distance between the intima and media complex from end-diastolic images. The maximum diameter was recorded. This process was repeated three times and the average of these measures used in the analysis. Carotid distension was determined by measuring the luminal diameter excursion from diastole to systole. The carotid distensibility coefficient (cDC), which reflects intrinsic vascular wall elasticity, was calculated using lumen diameter (D), change in lumen diameter (∆D) and change in blood pressures (∆P) in the equation ((2x∆DxD)+ ∆D^2^)/(∆PxD^2^) to reflect relative change in cross-sectional area per 10^-3^ kPa change in BP from systole to diastole^74^.

***MRI Pulse Wave Velocity***

2D velocity-encoded phase contrast MRI sequences were acquired through the proximal descending aorta (at the level of the pulmonary trunk) and the distal descending aorta (above the aortic bifurcation) in the plane perpendicular to the aortic long axis. A free-breathing retrospectively ECG-gated phase-contrast flow mapping sequence was used. A velocity-encoding gradient of 150 to 200 cm/s was applied in the through plane direction. Right brachial BP was recorded using a paediatric cuff immediately after the flow sequence acquisitions (In vivo MRI compatible patient monitor). Velocity flow curves were generated using open source software (Osirix). PWV (m/s) was calculated using Matlab (MathWorks, Natick, MA, USA) and the transit time method from Δd/Δt (Δd=distance, Δt=transit time of the systolic wavefront between the two flow acquisition sites).

***Arterial Distensibility***

Arterial distensibility was measured across the aortic root using high resolution SSFP cine acquisition, planed from long axis 3 chamber and left ventricular outflow tract (LVOT) views, across the aortic root. The slice was positioned perpendicular to the long axis of the ascending aorta at the level of the pulmonary trunk, 2-4 cm above the aortic valve to avoid distortion from aortic valve motion. Change in vessel lumen area across the cardiac cycle was measured on the bright blood SSFP cine images using automated segmentation. Vessel lumen area was measured at each of 25 phases across the cardiac cycle. Area measurement was repeated 20 times at each phase. Arterial distensibility was calculated from average values for maximum vessel area (Amax) and minimum vessel area (Amin), corresponding to end-systole and end-diastole respectively, and recorded pulse pressure (PP) (difference between systolic and diastolic BP). Arterial distensibility (10-3mmHg-1) = arterial strain / PP (where arterial strain = Amax - Amin / Amin).

***Flow mediated dilatation and reactive hyperaemia***

Measurements of flow mediated dilatation (FMD) and reactive hyperaemia (RH) were obtained from the right brachial artery, 5–10 cm above the antecubital fossa, using high-resolution ultrasound (Acuson Aspen Ultrasound System, Mountain View, CA, USA) with a linear probe, attached to a computer with MIA capture facilities, with the probe held in a stereotaxic clamp that allowed micrometer positional adjustment. First, the brachial artery was imaged, and this recorded for 1 minute (baseline). Brachial artery FMD was then induced by a 5 min inflation of a pneumatic cuff to 200 mmHg around the forearm immediately below the medial epicondyle, followed by rapid deflation using an automatic air regulator device. Brachial artery diameter was measured using edge detection software (Brachial Tools, MIA, IA, USA) from ECG-triggered ultrasound images captured at 3s intervals throughout the 11 min recording protocol. FMD was expressed as the maximum percentage change in vessel diameter from baseline. In parallel, the magnitude of the flow stimulus (Velocity Time Integral (VTI)) and HR were recorded continuously by pulse wave Doppler. RH is expressed as percent change in flow, from baseline to maximum flow within 15 s of cuff deflation (RH%) and was calculated as [((VTI peak x HR peak) – (VTI baseline x HR baseline)) / (VTI baseline x HR baseline)] x 100.

***Infinium HumanMethylation EPIC BeadChip array***

DNA methylation using the Infinium HumanMethylation EPIC BeadChip array was used to interrogate DNA methylation in 500 buffy coat samples. 1µg of the genomic DNA was treated with sodium bisulfite using Zymo EZ DNA Methylation-Gold kit (ZymoResearch, Irvine, California, USA, D5007) and processing of the Human MethylationEPIC (Infinium Methylation EPIC; Illumina, Inc. CA, USA) platform was carried out by the Centre for Molecular Medicine and Therapeutics (CMMT) (http://www.cmmt.ubc.ca). The Idat files were pre-processed in R statistical computing version 3.4.2 using Bioconductor package minfi^33, 34^. Fourteen samples with median absolute deviation (MAD) score < -5 were removed. One sample without data on smoking was removed. Using Meffil ^35^, five samples that showed sex discrepancy, three outlier samples from methylated vs. unmethylated comparison and seven outlier samples from control probes were also removed. A total of 470 umbilical cord tissue samples were used for subsequent analysis. Singular Value Decomposition Analysis (SVD) was applied to detect technical variation using CHAMP^36^. COMBAT from SVA^37^ was applied to remove the slide effect. BMIQ normalization method from Bioconductor package CHAMP^36^ was applied on beta value. The following probes were discarded: those with a detection p-value above 0.01 in one or more samples (N= 10685); a beadcount <3 in at least 5% of samples (N=883); non-CG probes (N=2860); probes located on SNPs (N=77657); probes on the X or Y chromosome (N=17197); multiple site reactive probes and probes aligned to multiple locations were also removed (N= 43301)^38^. The remaining 713508 probes were used in the analysis.

To adjust for differences in cellular heterogeneity, a reference-based prediction of the cell composition was carried out using the algorithm by Houseman et al.^39^ and the FlowSorted.CordBlood.450k package in R which utilizes the reference for cord blood cell compositions estimated by Andrews and Bakulski^40^. Regression models using limma^41^ were run with methylation as the outcome variable. All models included the following as covariates: maternal smoking, child’s sex, age at measurements, batch effect, position on chip and the predicted values for B-cell, CD4 T-cell, CD8 T-cell, monocyte, natural killer cell and nucleated red blood cell counts. The analysis was controlled for multiple testing with the Benjamini-Hochberg adjustment for false discovery rate. Methylation QTL (meQTL) analysis was carried out using the GEM package in R^42^. A MAF cut off of 0.05 was applied to the results. Multivariate analysis describing the association between blood cell components and technical variable on aPWV is shown in Supplementary Table 9.

**table S1a. Cohort characteristics for participants with and without methylation data**

|  | **Maternal measures** | | | |
| --- | --- | --- | --- | --- |
| **cohort characteristic** | **participants with DNA methylation data** | | **participants without DNA methylation data** | |
|  | **group/number** | **N % or median (5th, 95th percentile)** | **group/number** | **N (%) or median (5th, 95th percentile)** |
| Ethnicity | White | 470 (100%) | White | 11,342 (93.9%) |
|  | Other | 0 (0%) | Other | 743 (6.1%) |
| Smoking in pregnancy | no | 407 (86.6%) | No | 2,131 (83.1%) |
|  | yes | 63 (13.4%) | Yes | 433 (16.9%) |
| Women's pre-pregnancy BMI, kg/m^2^ | 470 | 24.1 (19.6, 35.1) | 11944 | 24.1 (19.3, 35.5) |
| Pregnancy weight gain, kg | 448 | 11.7 (3.9, 23.3) | 2015 | 11.9 (2.4, 22.9) |
| Early pregnancy: Oily fish portions/week | 386 | 0.5 (0.0, 4.5) | 1884 | 0.5 (0.0, 1.5) |
| Late pregnancy: Oily fish portions/week | 463 | 0.5 (0.0, 4.5) | 2186 | 0.5 (0.0, 4.5) |
| Early pregnancy: serum Vitamin B12 (pg/ml) | 367 | 370.5 (194.4, 605.2) | 1677 | 361.1 (195.7, 651.9) |
| Late pregnancy: serum Vitamin B12 (pg/ml) | 418 | 166.0 (94.0, 286.0) | 1668 | 160.0 (82.0, 305.0) |
| Early pregnancy: serum Vitamin D (nmol/l) | 372 | 63.5 (25.5, 110.0) | 1688 | 59.8 (24.5, 105.5) |
| Late pregnancy: serum Vitamin D (nmol/l) | 443 | 65.7 (28.0, 124.0) | 1889 | 57.0 (24.1, 119.0) |
| Early pregnancy: plasma EPA (μg/ml) | 316 | 13.0 (5.9, 30.0) | 690 | 14.2 (6.5, 29.2) |
| Late pregnancy: plasma EPA (μg/ml) | 377 | 5.3 (2.1, 15.7) | 1400 | 5.2 (2.0, 13.6) |
| Early pregnancy: plasma DHA (μg/ml) | 316 | 80.3 (46.8, 135.2) | 690 | 82.6 (48.8, 135.3) |
| Late pregnancy: plasma DHA (μg/ml) | 377 | 54.6 (28.0, 107.8) | 1400 | 53.8 (25.1, 103.6) |
| Pregnancy-induced high blood pressure | no | 447 (95.1%) | no | 2524 (93.7%) |
|  | yes | 23 (4.9%) | yes | 171 (6.3%) |
| pre-eclampsia | no | 465 (98.9%) | no | 2605 (96.8%) |
|  | yes | 5 (1.1%) | yes | 85 (3.2%) |

**table S1b.**

|  | **Child measures** | | | |
| --- | --- | --- | --- | --- |
| **cohort characteristic** | **participants with DNA methylation data** | | **participants without DNA methylation data** | |
|  | **group/number** | **N % or median (5th, 95th percentile)** | **Group/number** | **N (%) or median (5th, 95th percentile)** |
| Sex | Male | 241 (51.6%) | Male | 1,392 (51.8%) |
|  | Female | 226 (48.4%) | Female | 1,294 (48.2%) |
| Arterial distensibility (10-3 mmHg-1) | 186 | 11.1 (5.9, 19.2) | 119 | 10.1 (5.9, 18.7) |
| MRI descending aorta pulse wave velocity (m/s) | 210 | 3.4 (2.8, 4.4) | 127 | 3.4 (2.8, 4.6) |
| Heart rate (beats per minute) | 210 | 80.0 (64.6, 97.8) | 129 | 81.4 (65.1, 101.5) |
| Age at MRI measurements (years) | 210 | 9.5 (9.1, 9.9) | 129 | 9.4 (9.1, 9.9) |
| Carotid distensibility coefficient (mm/10-3 kPa) | 385 | 62.3 (37.1, 109.0) | 533 | 63.2 (35.3, 103.9) |
| Carotid intima-media thickness maximum (mm) | 393 | 0.5 (0.4, 0.6) | 549 | 0.5 (0.4, 0.6) |
| Systolic blood pressure (mmHg) | 386 | 105.0 (91.0, 120.0) | 536 | 105.0 (93.0, 123.0) |
| Diastolic blood pressure (mmHg) | 386 | 55.0 (44.0, 69.0) | 536 | 55.0 (44.0, 70.0) |
| Pulse Pressure (mmHg) | 386 | 50.0 (37.0, 66.0) | 536 | 50.0 (35.0, 66.0) |
| Age at IMTDC measurement (years) | 393 | 9.2 (8.9, 9.6) | 549 | 9.1 (8.8, 9.7) |
| Flow mediated dilatation (%) | 297 | 6.7 (1.4, 13.6) | 400 | 6.2 (1.7, 14.1) |
| Age at FMD measurement (years) | 297 | 9.2 (8.9, 9.6) | 400 | 9.1 (8.8, 9.7) |
| Reactive hyperaemia (%) | 297 | 546.8 (239.8, 982.1) | 393 | 526.5 (222.8, 1027.4) |
| Age at reactive hyperaemia measurement (years) | 297 | 9.21 (8.9,9.7) | 393 | 9.1 (8.8, 9.7) |

**table S2a. Significant associations between cord blood DNA methylation and measures of cardiovascular structure and stiffness at age 8-9 years in SWS children, adjusted for further factors.** All regression models included maternal smoking, child’s sex, age at measurements, batch effect, and position on chip and the predicted values for B-cells, CD4 T-cells, CD8 T-cells, monocytes, natural killer cells and nucleated red blood cell composition. Model 1 is additionally adjusted for child’s height and BMI at 8-9 years; Model 2 is additionally adjusted for child’s height, BMI at 8-9 years, maternal age at birth, maternal educational attainment and parity. Model 3 is additionally adjusted for pregnancy-induced hypertension and pre-eclampsia.

| **Measure of cardiovascular health ( 8-9 years)** | **gene region (CpG) measured at birth** | **model 1** | | **model 2** | | **model 3** | |
| --- | --- | --- | --- | --- | --- | --- | --- |
|  |  | **β (95% CI)** | **P-Value** | **β (95% CI)** | **P-Value** | **β (95% CI)** | **P-Value** |
| Descending aorta pulse wave velocity (m/s) | PPM1D (cg20793626) | -0.06(-0.09,-0.03) | 3.8E-04 | -0.06(-0.09,-0.03) | 5.3E-04 | -0.06(-0.09,-0.02) | 8.0E-04 |
|  | SYBU (cg21851496) | -0.03(-0.05,-0.01) | 1.4E-03 | -0.03(-0.05,-0.01) | 1.4E-03 | -0.03(-0.05,-0.01) | 1.8E-03 |
|  | intergenic (cg25721132) | 0.17(0.08,0.25) | 1.9E-04 | 0.17(0.08,0.26) | 2.2E-04 | 0.17(0.08,0.26) | 1.3E-04 |
|  | BTRC (cg19669439) | 0.25(0.14,0.36) | 2.2E-05 | 0.24(0.13,0.36) | 6.3E-05 | 0.25(0.14,0.36) | 2.4E-05 |
|  | RAPGEF6 (cg10878312) | 0.16(0.08,0.25) | 2.0E-04 | 0.17(0.08,0.26) | 1.9E-04 | 0.17(0.08,0.25) | 1.5E-04 |
|  | intergenic (cg09432349) | -0.09(-0.14,-0.05) | 5.5E-05 | -0.09(-0.14,-0.05) | 8.3E-05 | -0.1(-0.14,-0.05) | 1.5E-05 |
|  | EIF5A (cg24214214) | 0.19(0.09,0.28) | 2.0E-04 | 0.18(0.09,0.28) | 3.3E-04 | 0.2(0.1,0.29) | 6.1E-05 |
|  | intergenic (cg05131620) | 0.03(0.009,0.04) | 2.6E-03 | 0.03(0.01,0.05) | 1.1E-03 | 0.03(0.01,0.04) | 2.4E-03 |
|  | intergenic (cg26499089) | -0.07(-0.11,-0.04) | 4.8E-05 | -0.08(-0.11,-0.04) | 6.8E-05 | -0.07(-0.11,-0.04) | 5.6E-05 |
|  | HDGF2 (cg05522885) | 0.1(0.05,0.15) | 8.3E-05 | 0.1(0.05,0.16) | 1.0E-04 | 0.1(0.05,0.15) | 1.2E-04 |
|  | MLPH (cg01504215) | 0.07(0.04,0.1) | 9.0E-05 | 0.07(0.04,0.1) | 1.2E-04 | 0.07(0.03,0.1) | 1.2E-04 |
|  | CHD1L (cg16525692) | 0.2(0.1,0.3) | 7.3E-05 | 0.2(0.11,0.3) | 8.4E-05 | 0.19(0.1,0.29) | 1.2E-04 |
|  | BIN3 (cg17670421) | 0.07(0.04,0.11) | 3.3E-05 | 0.07(0.04,0.11) | 3.3E-05 | 0.08(0.05,0.11) | 3.9E-06 |
|  | NTRK3 (cg08921491) | -0.07(-0.1,-0.03) | 8.5E-05 | -0.06(-0.1,-0.03) | 1.5E-04 | -0.07(-0.1,-0.04) | 3.3E-05 |
|  | PPT2 (cg08509237) | 0.2(0.12,0.29) | 8.5E-06 | 0.21(0.12,0.3) | 8.0E-06 | 0.21(0.12,0.3) | 4.3E-06 |
|  | C5orf66 (cg14277284) | -0.07(-0.11,-0.03) | 5.4E-04 | -0.07(-0.11,-0.03) | 8.4E-04 | -0.07(-0.11,-0.03) | 5.6E-04 |
| Arterial distensibility (10^-3^ mmHg^-1^) | NLRP11 (cg15304800) | 0.31(0.16,0.46) | 1.1E-04 | 0.31(0.15,0.46) | 1.8E-04 | 0.3(0.14,0.45) | 2.5E-04 |
| Pulse Pressure (mmHg) | ADGRG5 (cg21194844) | -1.14(-1.75,-0.53) | 2.7E-04 | -1.14(-1.76,-0.51) | 3.9E-04 | -1.25(-1.88,-0.62) | 1.2E-04 |
|  | intergenic (cg07652189) | -0.67(-1.13,-0.22) | 4.1E-03 | -0.7(-1.17,-0.24) | 3.3E-03 | -0.89(-1.36,-0.42) | 2.3E-04 |

**table S2b. Significant associations between cord blood DNA methylation and general measures of cardiovascular health at age 8-9 years in SWS children, adjusted for further factors.** All regression models included maternal smoking, child’s sex, age at measurements, batch effect, and position on chip and the predicted values for B-cells, CD4 T-cells, CD8 T-cells, monocytes, natural killer cells and nucleated red blood cell composition. Model 1 is additionally adjusted for child’s height and BMI at 8-9 years; Model 2 is additionally adjusted for child’s height, BMI at 8-9 years, maternal age at birth, maternal educational attainment and parity. Model 3 is additionally adjusted for pregnancy-induced hypertension and pre-eclampsia.

| **Measure of cardiovascular health ( 8-9 years)** | **gene region (CpG) measured at birth** | **model 1** | | **model 2** | | **model 3** | |
| --- | --- | --- | --- | --- | --- | --- | --- |
|  |  | **β (95% CI)** | **P-Value** | **β (95% CI)** | **P-Value** | **β (95% CI)** | **P-Value** |
| Heart rate (mean beats per minute) | C6orf108 (cg09606840) | 0.73(0.42,1.05) | 9.8E-06 | 0.75(0.42,1.07) | 1.0E-05 | 0.71(0.4,1.02) | 1.6E-05 |
| Diastolic blood pressure (mmHg) | intergenic (cg24745895) | 1.76(1.03,2.49) | 3.4E-06 | 1.68(0.94,2.42) | 1.1E-05 | 1.71(0.97,2.45) | 8.5E-06 |
| Systolic blood pressure (mmHg) | CHN2 (cg14407341) | 2.46(1.15,3.77) | 2.8E-04 | 2.24(0.88,3.59) | 1.3E-03 | 2.96(1.55,4.38) | 5.0E-05 |
|  | CTDSP2 (cg05902531) | 3.79(2.16,5.43) | 7.6E-06 | 3.88(2.22,5.54) | 6.4E-06 | 4.61(2.85,6.37) | 4.6E-07 |
|  | intergenic (cg12256233) | -0.43(-0.62,-0.25) | 5.0E-06 | -0.43(-0.62,-0.25) | 5.3E-06 | -0.48(-0.68,-0.28) | 3.2E-06 |

**table S3. Associations between maternal blood fatty acid levels during pregnancy with dmCpG sites associated with the child’s aortic PWV.** Associations between eicosapentaenoic acid (EPA) and docosahexaenoic acid (DHA) measured in plasma (ug/ml) in early (n = 316) and late pregnancy (n = 377) and methylation of cg probes associated with aortic PWV at 8-9years. Multivariate analysis correcting for array batch, position, blood cell components (Bcell CD4T CD8T Mono NK nRBC), sex of child, smoking in pregnancy. PUFA data was log-transformed to correct for a non-normal distribution.

**(A) Measures in Early Pregnancy**

| **gene region (CpG) measured at birth** | **Plasma EPA (20 5n3)** | | | **Plasma DHA (22 6n3)** | | |
| --- | --- | --- | --- | --- | --- | --- |
|  | **regression with  non transformed data** | | **after log transformation of variable** | **regression with  non transformed data** | | **after log transformation of variable** |
|  | **β (95% CI)** | **P-Value** | **P-Value** | **β (95% CI)** | **P-Value** | **ln P-Value** |
| PPM1D (cg20793626) | 0.03(6e-04,0.06) | **0.05** | 0.06 | 0.01(0.00302,0.02) | **7.34E-03** | 0.05 |
| SYBU (cg21851496) | -0.01(-0.06,0.04) | 0.69 | 0.21 | -0.00794(-0.02,0.0067) | 0.29 | 0.33 |
| intergenic (cg25721132) | 0.001(-0.01,0.01) | 0.81 | 0.71 | -0.00125(-0.0044,0.0019) | 0.44 | 0.56 |
| BTRC (cg19669439) | 0.005(-0.004,0.01) | 0.26 | 0.16 | 0.00096(-0.00157,0.0035) | 0.46 | 0.54 |
| RAPGEF6 (cg10878312) | 0.005(-0.006,0.02) | 0.37 | 0.38 | -0.00066(-0.00397,0.0026) | 0.70 | 0.77 |
| intergenic (cg09432349) | -0.006(-0.03,0.02) | 0.56 | 0.38 | 6e-04(-0.00553,0.0067) | 0.85 | 0.84 |
| EIF5A (cg24214214) | -0.001(-0.01,0.01) | 0.80 | 0.75 | -0.00182(-0.00493,0.0013) | 0.25 | 0.12 |
| intergenic (cg05131620) | 0.02(-0.04,0.09) | 0.47 | 0.41 | 0.00423(-0.01,0.02) | 0.66 | 0.86 |
| intergenic (cg26499089) | 0.008(-0.02,0.03) | 0.56 | 0.57 | 0.00101(-0.00647,0.0085) | 0.79 | 0.94 |
| HDGF2 (cg05522885) | -0.004(-0.02,0.01) | 0.69 | 0.88 | -0.00132(-0.00659,0.0039) | 0.62 | 0.67 |
| MLPH (cg01504215) | 0.009(-0.02,0.04) | 0.57 | 0.92 | 0.00339(-0.00509,0.01) | 0.43 | 0.31 |
| CHD1L (cg16525692) | 0.007(-0.002,0.02) | 0.14 | 0.16 | -0.0016(-0.00436,0.0011) | 0.25 | 0.57 |
| BIN3 (cg17670421) | -0.004(-0.03,0.02) | 0.77 | 0.90 | 0.00205(-0.00602,0.01) | 0.62 | 0.21 |
| NTRK3 (cg08921491) | 0.01(-0.02,0.04) | 0.53 | 0.22 | 0.0088(-0.00015,0.02) | 0.06 | 0.48 |
| PPT2 (cg08509237) | -0.0006(-0.01,0.01) | 0.90 | 0.91 | -0.0011(-0.0039,0.0017) | 0.44 | 0.20 |
| C5orf66 (cg14277284) | -0.007(-0.03,0.02) | 0.57 | 0.68 | -0.0025(-0.00957,0.0046) | 0.49 | 0.83 |

**(B) Measures in Late Pregnancy**

| **gene region (CpG) measured at birth** | **Plasma EPA (20 5n3)** | | | **Plasma DHA (22 6n3)** | | |
| --- | --- | --- | --- | --- | --- | --- |
|  | **regression with  non-transformed data** | | **after log transformation of variable** | **regression with  non-transformed data** | | **after log transformation of variable** |
|  | **β (95% CI)** | **P-Value** | **P-Value** | **β (95% CI)** | **P-Value** | **ln P-Value** |
| PPM1D (cg20793626) | -0.00383(-0.04,0.03) | 0.85 | 0.81 | 0.00649(-0.0022,0.02) | 0.14 | 0.65 |
| SYBU (cg21851496) | -0.05(-0.12,0.02) | 0.16 | 0.11 | 0.001(-0.02,0.02) | 0.90 | 0.50 |
| intergenic (cg25721132) | -0.00063(-0.02,0.01) | 0.93 | 0.79 | 0.00014(-0.00325,0.0035) | 0.94 | 0.76 |
| BTRC (cg19669439) | 0.00607(-0.00511,0.02) | 0.29 | 0.15 | 0.00094(-0.00163,0.0035) | 0.47 | 0.38 |
| RAPGEF6 (cg10878312) | 0.01(-0.00356,0.03) | 0.13 | 0.19 | -5e-05(-0.00355,0.0034) | 0.98 | 0.32 |
| intergenic (cg09432349) | 0.00615(-0.02,0.03) | 0.67 | 0.38 | -0.00725(-0.01,-9e-04) | **0.02** | 0.09 |
| EIF5A (cg24214214) | 0.00524(-0.00886,0.02) | 0.47 | 0.14 | 0.00117(-0.002,0.0043) | 0.47 | 0.73 |
| intergenic (cg05131620) | -0.01(-0.1,0.07) | 0.73 | 0.33 | -0.02(-0.04,7e-04) | 0.06 | 0.06 |
| intergenic (cg26499089) | 0.00765(-0.03,0.04) | 0.68 | 0.39 | 0.00417(-0.00413,0.01) | 0.33 | 0.42 |
| HDGF2 (cg05522885) | 0.00239(-0.02,0.03) | 0.85 | 0.63 | -0.00154(-0.00724,0.0042) | 0.60 | 0.95 |
| MLPH (cg01504215) | -0.02(-0.06,0.02) | 0.35 | 0.44 | 0.00014(-0.00874,0.009) | 0.98 | 0.29 |
| CHD1L (cg16525692) | -0.00067(-0.01,0.01) | 0.92 | 0.68 | -0.00273(-0.00559,1e-04) | 0.06 | 0.19 |
| BIN3 (cg17670421) | 0.01(-0.03,0.05) | 0.54 | 0.91 | 0.00084(-0.00784,0.0095) | 0.85 | 0.71 |
| NTRK3 (cg08921491) | 0.03(-0.0084,0.07) | 0.12 | 0.29 | 0.00452(-0.00457,0.01) | 0.33 | 0.82 |
| PPT2 (cg08509237) | -0.00569(-0.02,0.0077) | 0.41 | 0.15 | -0.00363(-0.00665,-6e-04) | **0.02** | **0.03** |
| C5orf66 (cg14277284) | 0.01(-0.02,0.04) | 0.49 | 0.50 | 0.00019(-0.00724,0.0076) | 0.96 | 0.79 |

**table S4. Interaction between identified maternal factors and dmCpG sites.** Testing for interaction between CpG methylation and associated maternal factors, in a model adjusting for the main effect of CpG methylation, main effect of maternal factor, as well as array batch, position, blood cell components (Bcell CD4T CD8T Mono NK nRBC), sex of child, smoking in pregnancy and age of measurement, with aPWV as the outcome.

| **gene region (CpG) measured at birth** | **n** | **maternal factor** | **CpG*maternal factor** | |
| --- | --- | --- | --- | --- |
|  |  |  | **β (95% CI)** | **P-Value** |
| HDGF2 (cg05522885) | 210 | Women's pre-pregnancy BMI (kg/m2) | -0.003 (-0.01,0.01) | 0.530 |
| PPT2 (cg08509237) | 173 | EP: Oily fish (portions/week) | 0.075 (0.002,0.15) | 0.045 |
| PPT2 (cg08509237) | 209 | LP: Oily fish (portions/week) | 0.018 (-0.08,0.12) | 0.730 |
| Intergenic (cg09432349) | 196 | Pregnancy weight gain (kg) | 0.002 (0,0.01) | 0.524 |
| BTRC (cg19669439) | 173 | EP: Oily fish (portions/week) | 0.025 (-0.12,0.16) | 0.727 |
| PPM1D (cg20793626) | 196 | Pregnancy weight gain (kg) | -0.005 (-0.01,0) | 0.113 |
| EIF5A (cg24214214) | 196 | Pregnancy weight gain (kg) | 0.013 (0,0.03) | 0.157 |
| Intergenic (cg26499089) | 169 | EP: serum Vit D (nmol/L) | 0.001 (0,0) | 0.103 |
| intergenic (cg25721132) | 210 | Smoking in pregnancy | 0.184 (-0.03,0.4) | 0.089 |

**table S5. Gene Environment Methylation analysis identifying SNP/CpG associations.** Associations between genotype and dmCpG sites significantly associated with aPWV.

| **CpG** | **SNP** | **reference allele** | **alternative allele** | **SNP Chr.** | **SNP position** | **CpG correlation** | **p value** | **FDR** |
| --- | --- | --- | --- | --- | --- | --- | --- | --- |
| PPM1D (cg20793626) | rs1864954 | A | G | 5 | 168123313 | -0.14 | 3.64E-09 | 0.005 |
|  | rs16871449 | C | A | 5 | 41523175 | -0.04 | 1.02E-08 | 0.012 |
|  | rs3923066 | C | T | 8 | 56611628 | -0.23 | 5.57E-08 | 0.042 |
|  | rs10105904 | T | C | 8 | 56639770 | -0.17 | 6.03E-08 | 0.045 |
| EIF5A (cg24214214) | rs2048806 | A | G | 3 | 55043070 | 0.05 | 1.16E-08 | 0.013 |
| NTRK3 (cg08921491) | rs17327937 | T | G | 4 | 93872906 | 0.12 | 3.10E-08 | 0.027 |
| C5orf66 (cg14277284) | rs11103699 | G | A | 9 | 138017196 | -0.18 | 6.70E-08 | 0.048 |
| intergenic (cg05131620) | rs2540709 | C | T | 16 | 55473882 | -0.59 | 8.53E-10 | 0.003 |
|  | rs2576542 | A | G | 16 | 55479952 | -0.50 | 8.53E-10 | 0.003 |
|  | rs1420227 | T | C | 16 | 55465544 | -0.49 | 3.29E-12 | 1.93E-05 |
|  | rs12924764 | G | A | 16 | 55498049 | -0.48 | 2.23E-08 | 0.037 |
|  | rs2160279 | T | C | 16 | 55470889 | -0.45 | 1.02E-10 | 4.97E-04 |
|  | rs8045690 | T | C | 16 | 55471946 | -0.35 | 2.21E-25 | 8.35E-18 |
|  | rs11644688 | G | A | 16 | 55475736 | -0.34 | 8.44E-22 | 1.86E-14 |
|  | rs1816594 | C | T | 16 | 55489908 | -0.34 | 1.31E-08 | 0.026 |
|  | rs1005913 | T | G | 16 | 55504521 | -0.34 | 7.27E-13 | 4.92E-06 |
|  | rs16955080 | C | T | 16 | 55463202 | 0.33 | 1.34E-17 | 1.96E-10 |
|  | rs11643666 | G | A | 16 | 55490089 | -0.26 | 1.11E-17 | 1.72E-10 |
|  | rs1610101 | T | C | 16 | 55460729 | 0.33 | 3.00E-10 | 0.001 |

**table S6. Associations between genetic variants and dmCpG sites associated with the child’s aortic PWV.** Associations between genetic variation identified through GEM analysis and CpG sites associated with aPWV. All multivariate regression analyses are adjusted for array batch, position, blood cell components (Bcell CD4T CD8T Mono NK nRBC), sex of child and smoking in pregnancy. For analysis of genetic effect on PPM1D (cg20793626) and intergenic (cg05131620) principal components (PCs) were generated from SNPs associated with the CpG site. For PPM1D (cg20793626), principal component analysis (PCA) was performed on four SNPs: rs1864954, rs16871449, rs3923066, rs10105904; PC1 and PC2 explained 78% of the variance. For intergenic (cg05131620), PCA was performed on twelve SNPs listed in Supplementary Table 3; PC1 and PC2 explained 76% of the variance.

| **CpG site** | **covariate** | | **β (95% CI)** | **P-Value** | **Adj. R-squared for model** |
| --- | --- | --- | --- | --- | --- |
| EIF5A (cg24214214) | rs2048806 genotype | AA | (reference) | - | 3.0% |
|  |  | AG | 0.120 (-0.06,0.301) | 0.192 |  |
|  |  | GG | -0.203 (-0.403,-0.002) | 0.047 |  |
|  | Adj. R-squared for model containing: array batch, position, blood cell components, sex of child and smoking in pregnancy only | | | | -0.1% |
| NTRK3 (cg08921491) | rs17327937 genotype | TT | (reference) | - | 14.6% |
|  |  | TG | 0.197 (-0.345,0.739) | 0.475 |  |
|  |  | GG | -3.142 (-5.269,-1.014) | 0.004 |  |
|  | Adj. R-squared for model containing: array batch, position, blood cell components, sex of child and smoking in pregnancy only | | | | 13.2% |
| C5orf66 (cg14277284) | rs11103699 genotype | GG | (reference) | - | 9.4% |
|  |  | GA | -0.303 (-0.699,0.093) | 0.134 |  |
|  |  | AA | -2.765 (-4.021,-1.508) | 1.89E-05 |  |
|  | Adj. R-squared for model containing: array batch, position, blood cell components, sex of child and smoking in pregnancy only | | | | 5.6% |
| intergenic (cg05131620) | PCA analysis | PC1 | -1.397 (-1.499,-1.295) | 2.00E-16 | 77.6% |
|  |  | PC2 | 0.648 (0.519,0.777) | 2.00E-16 |  |
|  | Adj. R-squared for model containing: array batch, position, blood cell components, sex of child and smoking in pregnancy only | | | | 34.9% |
| PPM1D (cg20793626) | PCA analysis | PC1 | -0.145 (-0.273,-0.017) | 0.026 | 13.8% |
|  |  | PC2 | 0.41 (0.237,0.584) | 4.19E-06 |  |
|  | Adj. R-squared for model containing: array batch, position, blood cell components, sex of child and smoking in pregnancy only | | | | 8.5% |

**table S7. Influence of identified SNPs on associations with aPWV.** (A) Associations between genetic variants identified by GEM analysis and aPWV at 8-9yrs. Model adjusted for: sex of child, smoking in pregnancy and age at measurement. (B) Comparison of associations between CpG sites associated with aPWV at 8-9yrs. after taking account of identified genetic variants, adjusting for array batch, position, blood cell components (B cell, CD4T, CD8T, Mono, NK, nRBC), sex of child, smoking in pregnancy and age of measurement. Analysis was carried out on a subset of samples for which both genetic and epigenetic data was available. (C) Testing for interaction between CpG methylation and the most significantly associated SNP, in a model adjusting for the main effect of CpG methylation, SNP, as well as array batch, position, blood cell components (B cell, CD4T, CD8T, Mono, NK, nRBC), sex of child, smoking in pregnancy and age of measurement.

**(A)**

| **CpG site** | **component** | **β (95% CI)** | **P-Value** |
| --- | --- | --- | --- |
| rs2048806 | AA | (reference) | - |
|  | AG | 0.06 (-0.1,0.24) | 0.44 |
|  | GG | -0.03 (-0.22,0.15) | 0.73 |
| rs17327937 | TT | (reference) | - |
|  | TG | -0.03 (-0.19,0.13) | 0.72 |
|  | GG | 0.55 (-0.1,1.22) | 0.10 |
| rs11103699 | GG | (reference) | - |
|  | GA | 0.03 (-0.13,0.2) | 0.67 |
|  | AA | 0.02 (-0.4,0.44) | 0.92 |
| PCA components for SNPs associated with cg05131620 | PC1 | -0.03 (-0.06,0) | 0.02 |
|  | PC2 | 0.03 (0,0.06) | 0.06 |
| PCA components for SNPs associated with PPM1D | PC1 | 0.03 (-0.01,0.07) | 0.19 |
|  | PC2 | -0.01 (-0.08,0.05) | 0.63 |

**(B)**

| **gene region (CpG) measured at birth** | **n** | **without adjustment for genetic variation** | | **with adjustment for genetic variation** | |
| --- | --- | --- | --- | --- | --- |
|  |  | **β (95% CI)** | **P-Value** | **β (95% CI)** | **P-Value** |
| PPM1D (cg20793626) | 206 | -0.05 (-0.09,-0.02) | 4.16E-04 | -0.06 (-0.1,-0.02) | 9.91E-04 |
| EIF5A (cg24214214) | 209 | 0.18 (0.09,0.28) | 1.78E-04 | 0.2 (0.1,0.31) | 2.29E-04 |
| Intergenic (cg05131620) | 206 | 0.03 (0.01,0.04) | 7.92E-04 | 0.03 (0.01,0.06) | 0.052 |
| NTRK3 (cg08921491) | 209 | -0.06 (-0.09,-0.03) | 5.22E-05 | -0.06 (-0.1,-0.03) | 4.28E-04 |
| C5orf66 (cg14277284) | 209 | -0.06 (-0.1,-0.02) | 1.17E-03 | -0.08 (-0.12,-0.03) | 5.66E-04 |

| **(C)** |  |  |  |  |
| --- | --- | --- | --- | --- |
| **gene region (CpG) measured at birth** | **SNP** | **CpG*SNP interaction** | **β (95% CI)** | **P-Value** |
| EIF5A (cg24214214) | rs2048806 | cg24214214*rs2048806 | -0.006 (-0.16,0.15) | 0.937 |
| NTRK3 (cg08921491) | rs17327937 | cg08921491*rs17327937 | 2.345 (-7.91,12.6) | 0.653 |
| C5orf66 (cg14277284) | rs11103699 | cg14277284*rs11103699 | 0.021 (-0.06,0.1) | 0.588 |
| PPM1D (cg20793626) | rs1864954 | cg14277284*rs1864954 | 0.021 (-0.06,0.1) | 0.588 |
| intergenic (cg05131620) | rs8045690 | cg05131620*rs8045690 | 0.721 (-2.31,3.76) | 0.640 |

**table S8. Multivariate linear regression showing estimated variance in aPWV explained by associated dmCpGs.** Multivariate regression analysis. For analysis without covariates methylation data was pre-adjusted to remove the slide effect.

Technical covariates adjusted for in linear regression: array batch, position, blood cell components (B cell, CD4T, CD8T, Mono, NK, nRBC counts). Physiological covariates: sex of child and smoking in pregnancy.

| **CpG site in model** | **CpG sites in model without covariates** | | **CpG sites in model alongside technical covariates** | | **CpG sites in model alongside technical and physiological covariates** | |
| --- | --- | --- | --- | --- | --- | --- |
|  | **β (95% CI)** | **p-value** | **β (95% CI)** | **p-value** | **β (95% CI)** | **p-value** |
| cg20793626 | -0.02 (-0.05,0) | 0.05 | -0.02 (-0.05,0) | 0.05 | -0.02 (-0.05,0) | 0.06 |
| cg21851496 | -0.02 (-0.04,-0.01) | 9.66E-04 | -0.03 (-0.04,-0.01) | 2.15E-05 | -0.03 (-0.05,-0.02) | 2.16E-06 |
| cg25721132 | 0.04 (-0.03,0.12) | 0.26 | 0.07 (0,0.15) | 0.08 | 0.06 (-0.01,0.14) | 0.13 |
| cg10878312 | 0.1 (0.02,0.18) | 8.31E-03 | 0.08 (0,0.15) | 0.04 | 0.08 (0,0.15) | 0.03 |
| cg09432349 | -0.01 (-0.05,0.01) | 0.22 | -0.04 (-0.08,0) | 1.35E-02 | -0.04 (-0.08,0) | 1.39E-02 |
| cg24214214 | 0.02 (-0.05,0.1) | 0.56 | 0.03 (-0.04,0.12) | 0.34 | 0.05 (-0.02,0.13) | 0.21 |
| cg05131620 | 0 (0,0.01) | 0.41 | 0 (0,0.02) | 0.17 | 0 (0,0.02) | 0.18 |
| cg26499089 | -0.02 (-0.06,0) | 0.07 | -0.03 (-0.06,0) | 1.48E-02 | -0.04 (-0.07,-0.01) | 7.12E-03 |
| cg05522885 | 0.04 (0,0.09) | 0.07 | 0.04 (0,0.09) | 0.06 | 0.05 (0,0.09) | 0.03 |
| cg01504215 | 0 (-0.01,0.03) | 0.54 | 0.02 (0,0.05) | 0.09 | 0.02 (0,0.05) | 0.10 |
| cg16525692 | 0.07 (-0.02,0.17) | 0.13 | 0.05 (-0.04,0.15) | 0.25 | 0.05 (-0.04,0.14) | 0.27 |
| cg17670421 | 0.07 (0.04,0.1) | 4.28E-07 | 0.06 (0.03,0.08) | 4.43E-05 | 0.06 (0.03,0.08) | 1.86E-05 |
| cg08921491 | -0.01 (-0.04,0.01) | 0.23 | -0.02 (-0.05,0) | 0.10 | -0.01 (-0.04,0) | 0.17 |
| cg08509237 | 0.01 (-0.06,0.1) | 0.65 | 0.03 (-0.04,0.11) | 0.37 | 0.04 (-0.03,0.13) | 0.24 |
|  |  |  |  |  |  |  |
| Adjusted R squared for model | 32.4% | | 42.8% | | 45.4% | |

**table S9. Multivariate linear regression models examining relationship between blood cell components and aPWV.** (A) Model with blood cell components and age at measurement, with aPWV as the outcome. (B) Blood cell components alongside other technical and physiological covariates, with aPWV as the outcome.

| **Basic model containing blood cell components** | |  | **model including technical covariates** |  |
| --- | --- | --- | --- | --- |
| **covariate in model** | **β (95% CI)** | **P-Value** | **β (95% CI)** | **P-Value** |
| CD4 T-cells | -0.528 (-2.05,1) | 0.50 | -0.642 (-2.24,0.95) | 0.43 |
| CD8 T-cells | 1.208 (-0.57,2.99) | 0.18 | 0.849 (-0.99,2.69) | 0.36 |
| B-cells | 0.114 (-2.7,2.93) | 0.94 | 1.177 (-1.82,4.17) | 0.44 |
| Monocytes | -2.453 (-5.57,0.67) | 0.12 | -2.712 (-5.93,0.51) | 0.10 |
| nucleated red blood cells | -0.658 (-1.88,0.57) | 0.29 | -0.336 (-1.65,0.98) | 0.61 |
| Natural killer cells | -2.432 (-7.89,3.02) | 0.38 | -2.827 (-8.38,2.73) | 0.32 |
| age at measurement | 0.092 (-0.21,0.39) | 0.54 | 0.148 (-0.17,0.46) | 0.35 |
| sex |  |  | -0.015 (-0.15,0.12) | 0.83 |
| smoking in pregnancy |  |  | 0.112 (-0.09,0.31) | 0.27 |
| position on array 2 |  |  | 0.088 (-0.18,0.36) | 0.52 |
| position on array 3 |  |  | -0.051 (-0.31,0.21) | 0.69 |
| position on array 4 |  |  | 0.193 (-0.08,0.46) | 0.16 |
| position on array 5 |  |  | 0.006 (-0.3,0.31) | 0.97 |
| position on array 6 |  |  | 0.298 (0.02,0.57) | 0.03 |
| position on array 7 |  |  | 0.13 (-0.15,0.41) | 0.36 |
| position on array 8 |  |  | 0.18 (-0.1,0.46) | 0.21 |
| Batch 2 |  |  | 0.003 (-0.19,0.19) | 0.97 |
| Batch 3 |  |  | -0.032 (-0.24,0.18) | 0.76 |
| Batch 4 |  |  | 0.091 (-0.14,0.32) | 0.43 |
| Batch 5 |  |  | 0.054 (-0.46,0.56) | 0.83 |
| Batch 6 |  |  | 0.023 (-0.25,0.3) | 0.87 |
| Batch 7 |  |  | -0.308 (-0.82,0.2) | 0.24 |

**DATA SUPPLEMENT – Figures and Tables**

**figure S1. Venn diagram showing common dmCpGs between different cardiovascular measurements.** (A). Overlap between dmCpG sites associated with structural and functional measures of cardiovascular function at FDR≤ 0.25. (B) Overlap between dmCpG sites associated with SBP, DBP and Pulse Pressure at FDR≤ 0.25.

**
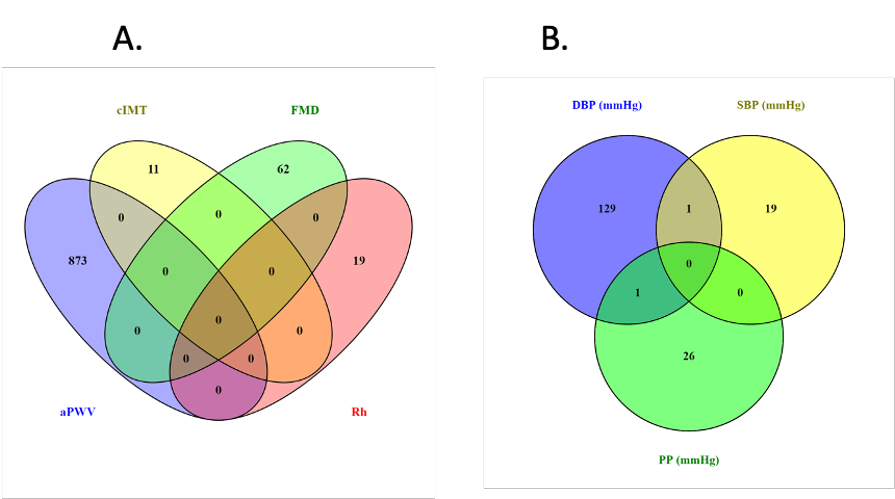
**

**figure S2.** **Protein-protein interaction (PPI) networks related to 8-9 year MRI PWV.** (A) PPI network (628 genes, ≤0.25 FDR), PPI enrichment p-value: 1.89x10^-11^. (B) Top five clusters generated using MCODE associated with a significant Gene Ontology (GO) term. (C) GO terms associated with identified clusters.

**
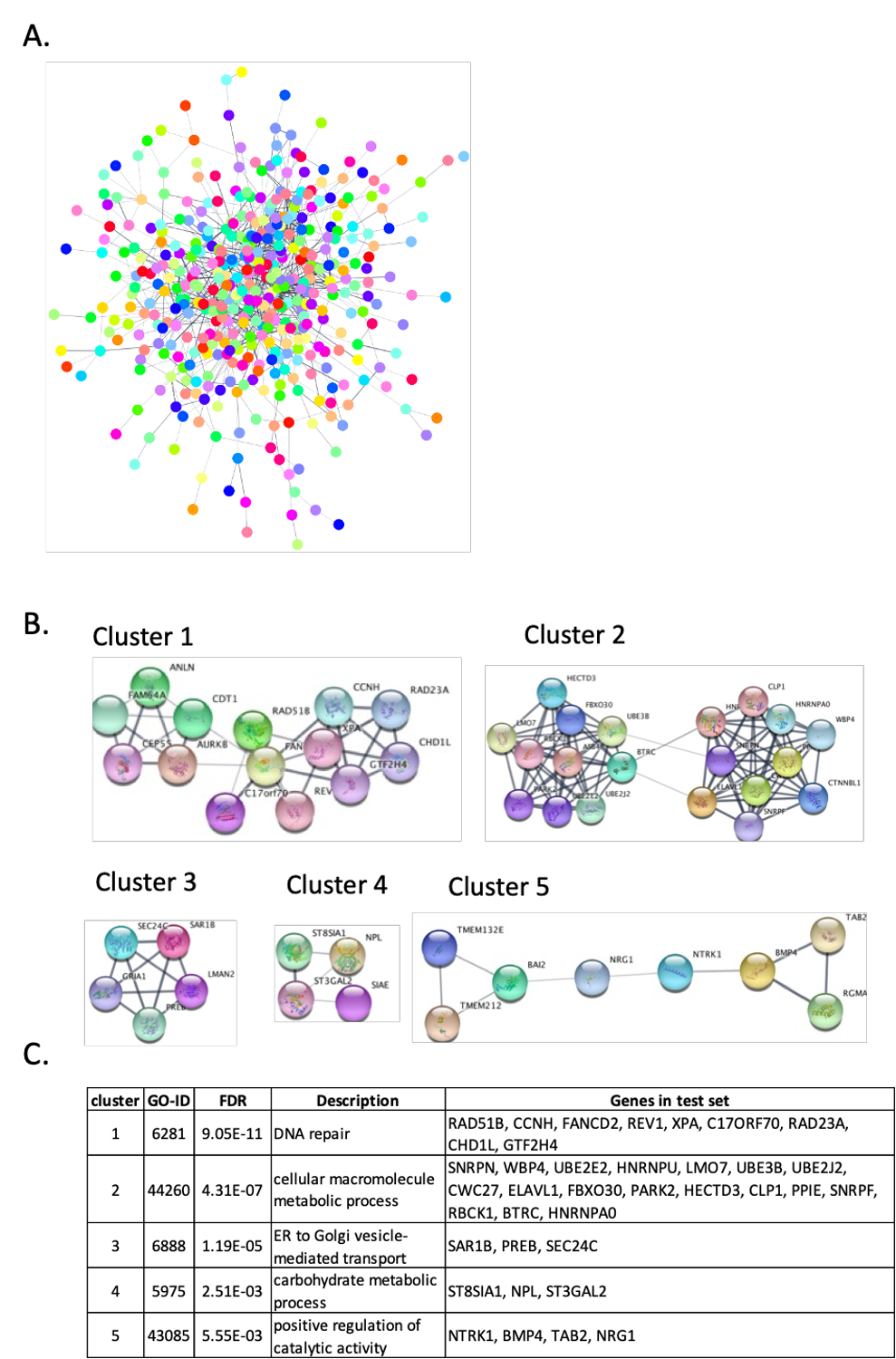
**

**figure S3. Associations between cg08509237 (PPT2) methylation and aPWV, stratified by oily fish consumption.** Scatter plot of cg08161364 % methylation against aPWV at 8-9 years, stratified by reported oily fish consumption in early pregnancy.

**
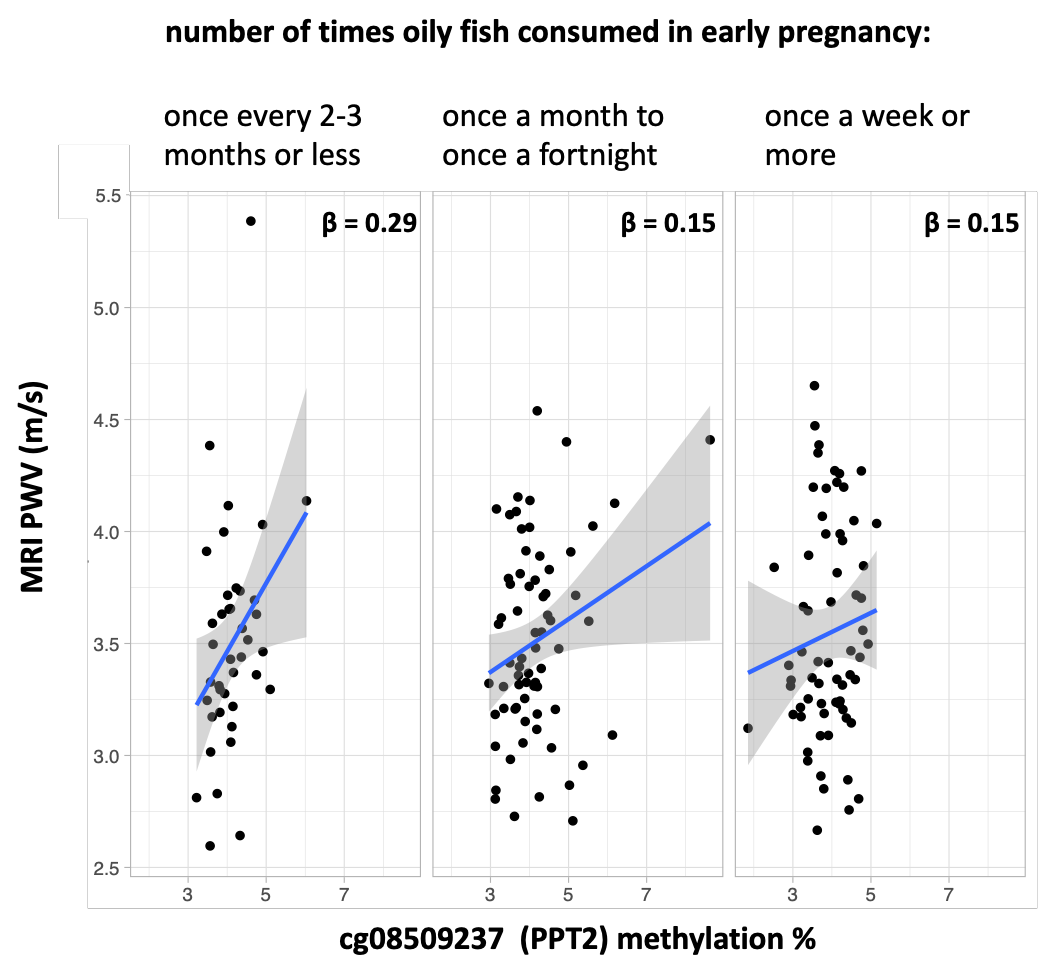
**
